# Metabolomics and transcriptomics reveal the mechanism of alkaloid synthesis in *Corydalis yanhusuo* bulbs

**DOI:** 10.1371/journal.pone.0304258

**Published:** 2024-05-23

**Authors:** Xiao Zhao, Yuan Pan, Jun Tan, Hui Lv, Yu Wang, Da-xia Chen

**Affiliations:** 1 Chongqing Academy of Chinese Materia Medica, Chongqing, China; 2 Chongqing College of Traditional Chinese Medicine, Chongqing, China; 3 Chongqing Engineering Research Center for Fine Variety Breeding Techniques of Chinese Materia Medica, Chongqing, China; 4 Chongqing Sub-Center of National Resource Center for Chinese Materia Medica, China Academy of Chinese Medical Science, Chongqing, China; Hainan University, CHINA

## Abstract

*Corydalis yanhusuo* W.T. Wang is a traditional herb. Benzylisoquinoline alkaloids (BIAs) are the main pharmacological active ingredients that play an important role in sedation, relieving pain, promoting blood circulation, and inhibiting cancer cells. However, there are few studies on the biosynthetic pathway of benzylisoquinoline alkaloids in *Corydalis yanhusuo*, especially on some specific components, such as tetrahydropalmatine. We carried out widely targeted metabolome and transcriptomic analyses to construct the biosynthetic pathway of benzylisoquinoline alkaloids and identified candidate genes. In this study, 702 metabolites were detected, including 216 alkaloids. Protoberberine-type and aporphine-type alkaloids are the main chemical components in *C*. *yanhusuo* bulbs. Key genes for benzylisoquinoline alkaloids biosynthesis, including *6-OMT*, *CNMT*, *NMCH*, *BBE*, *SOMT1*, *CFS*, *SPS*, *STOX*, *MSH*, *TNMT* and *P6H*, were successfully identified. There was no significant difference in the content of benzylisoquinoline alkaloids and the expression level of genes between the two suborgans (mother-bulb and son-bulb). The expression levels of BIA genes in the expansion stage (MB-A and SB-A) were significantly higher than those in the maturity stage (MB-C and SB-C), and the content of benzylisoquinoline alkaloids was consistent with the pattern of gene regulation. Five complete single genes were likely to encode the functional enzyme of CoOMT, which participated in tetrahydropalmatine biosynthesis in *C*. *yanhusuo* bulbs. These studies provide a strong theoretical basis for the subsequent development of metabolic engineering of benzylisoquinoline alkaloids (especially tetrahydropalmatine) of *C*. *yanhusuo*.

## Introduction

*Corydalis yanhusuo* W.T. Wang, commonly known as Yuanhu, is a Corydalis plant of the Papaveraceae family. As a traditional Chinese herbal medicine, *C*. *yanhusuo* bulb has the functions of promoting blood circulation, relieving pain and remaining calm [1]. At present, there are many studies about the extraction and identification of chemical components from *C*. *yanhusuo*, including fatty acids, amino acids, steroids, organic acids, sugars, trace elements and alkaloids [2]. Over 60 kinds of alkaloids were successfully extracted and identified in bulbs. Benzylisoquinoline alkaloids (BIAs) are regarded as the major pharmacological components [3–5] and can be divided into several categories, including protoberberine, aporphine, benzo[c]phenanthridine, phthalideisoquinoline and morphine alkaloids. Protoberberine-type and aporphine-type BIAs account for the largest proportion, but protopine-type and benzo[c]phenanthridine-type BIAs are also present in extracts of *C*. *yanhusuo* bulbs [6–8]. Tetrahydropalmatine (THP), corydaline, protoopioid, tetrahydroberberine and others were successfully extracted and identified in *C*. *yanhusuo* bulbs [9–14]. However, little attention has been given to BIAs biosynthesis in Corydalis plants, which has resulted in little knowledge about the bulbs of *C*. *yanhusuo*, such as glaucine [15], corybulbine [16] and dihydrosanguinarine [17]. Modern medical research shows that BIAs in *C*. *yanhusuo* have strong effects on analgesia, sedation, hypnosis and anti-anxiety [18, 19]. The analgesic potency of total alkaloids of *C*. *yanhusuo* can be up to 40% of that of morphine [2, 12], and tetrahydropalmatine (THP) has the strongest analgesic effect with a low addiction [13]. At present, many new studies showed that *C*. *yanhusuo* bulbs also have other broad pharmacological activities, such as anti-myocardial ischaemia, anti-gastric ulcer, antitumour cell, antioxidation and liver protection activities [20–22], which has attracted widespread attention by researchers.

Currently, with the rise of the herbal genomics and the combined analysis of multiple omics (transcriptomics, metabolomics), it has become a powerful tool for studying the secondary metabolic pathways and key genes of active ingredients in medicinal plants[23, 24]. Meanwhile, due to the transformation of biotechnology methods such as genetic engineering and metabolic engineering, the research on active ingredients has developed rapidly, providing new avenues for modern Chinese medicine research [25–27]. Research on *C*. *yanhusuo* has mainly focused on the extraction process and pharmacological effects of active components [2, 19], but less on the synthesis pathway of the main compounds and the expression and regulation of key functional genes. Fortunately, the synthesis pathway of BIAs has been well described in the *Opium poppy*, which is used by a model plant [20, 22, 28, 29]. All the final metabolites need to take tyrosine as the substrate and form (S)-reticuline through the common pathway [22]. (S)-reticuline is an important intermediate substance for the synthesis of downstream BIAs [30]. However, there are few studies about metabolic pathways and functional enzyme genes in *C*. *yanhusuo* bulbs [31]. Some key steps and genes in the BIAs pathway are still missing, which greatly hinders the development of molecular cloning and metabolic engineering. In this study, we collected bulbs from two developmental stages and two suborgan parts of *C*. *yanhusuo* based on widely targeted metabolome and transcriptomic sequencing, aiming to fully construct the BIAs metabolism pathway and identify key functional genes, providing an important theoretical basis for the synthesis and regulation of BIAs in *C*. *yanhusuo* bulbs.

## Materials and methods

### Initiation and development of the bulb of *Corydalis yanhusuo* W.T. Wang

In general, bulbs of *Corydalis yanhusuo* can be divided into "mother-bulb (MB)" and "son-bulb (SB)" according to different parts. The mother bulbs are formed by the degeneration and re-expansion of their original stem and are used as medicinal material in production. Son bulbs emerge from axillary buds on horizontally elongated rhizomes, of which the larger bulb can also be used as medicine, while the smaller bulb is reserved as a seed stem for "seed". In the process of field production, the seed stems are planted in late September of that year. After a winter of dormancy, the aboveground parts begin to grow and develop in February of the next year. The development period of the underground part is short, approximately 40 days [31], from mid-late March to the end of April. Generally, the growth and expansion period of the bulb is from mid-late March to the middle of April (approximately 30 days), and the maturity period of the bulb (the end of expansion) is from late April, when the aboveground part is withering (approximately 15 days).

### Plant material collection

This study did not contain participants, specimens or tissue samples of human beings or vertebrate animals, embryos or tissues:

1. We claim clearly that these locations or activities need no specific permission. The experimental site is a medicinal herb planting base of the Chongqing Academy of Chinese Materia Medica, where we have carried out related research on the cultivation of *Corydalis yanhusuo*.
2. We confirm that our studies did not relate to endangered or protected species.
3. We confirm that the authors received approval from the Chongqing Academy of Chinese Materia Medica to collect samples from the plants.

**Fig 1:**
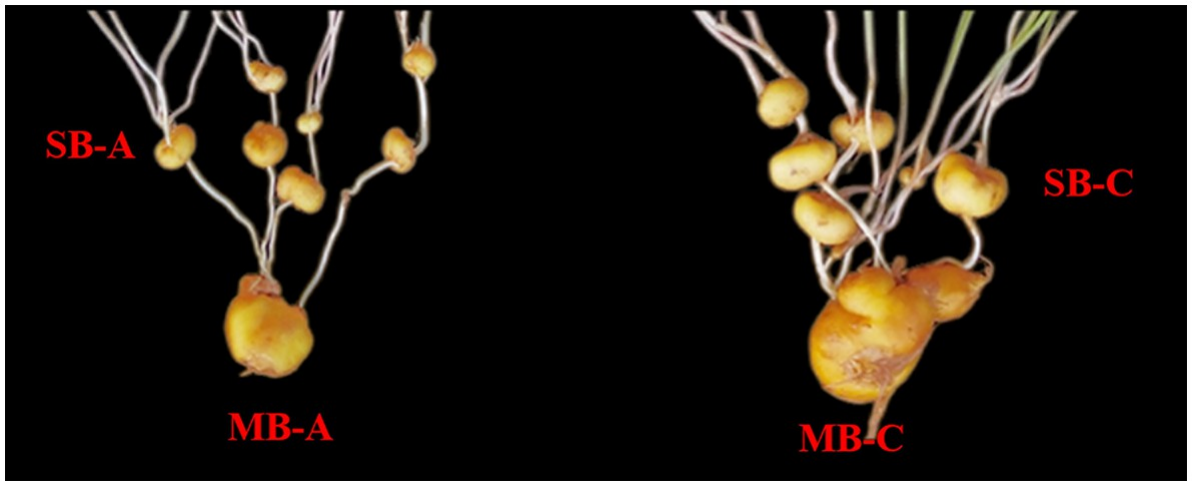
Different suborgan parts and development periods of *C*. *yanhusuo* bulb. MB-A: mother-bulb expansion period, SB-A: son-bulb expansion period, MB-C: mother-bulb maturity period, SB-C: son-bulb maturity period.

In this study, materials of *C*. *yanhusuo* bulbs were cultivated in the field, which was proposed and identified by Professor Da-xia Chen. The test site was located in Shizhu County, Chongqing, China (E108.096, N29.943), at an altitude of 1000 m. To ensure the consistency of sampling, we selected seed bulbs of the same size for planting and ensured that the environmental conditions, such as soil, fertilizer and water, were uniform. We sowed them at the end of September 2019. On April 6th (expansion period) and April 26th (maturity period) of the next year, samples of the "mother bulb" and "son bulb" of *C*. *yanhusuo* were collected (Fig 1). After washing, the samples were immediately placed into liquid nitrogen for quick freezing and then transferred to an ultralow temperature refrigerator for storage so that they could be used for metabolome and transcriptome analysis in the later stage.

### Metabolite extraction

After vacuum freeze-drying, the biological sample was crushed at 30 Hz for 1.5 minutes using a mixer (MM 400, Retsch) with zirconia beads. One hundred milligrams of lyophilized powder was accurately weighed, dissolved in 1.2 ml of 70% methanol solution, rotated every 30 minutes for 30 seconds, repeated 6 times, and placed in a refrigerator at 4 °C for 12 h. Finally, after centrifuging the solution at 12 000 rpm for 10 minutes, the extract was filtered (SCAA-104, 0.22 μm pore size; ANPEL, Shanghai, China, http://www.anpel.com.cn/) to perform UPLC–MS/MS analysis.

### Metabolite analysis by liquid chromatography-electrospray ionization-tandem mass spectrometry (UPLC-ESI-Q TRAP-MS/MS)

LIT and triple quadrupole (QQQ) scans were acquired on a triple quadrupole-linear ion trap mass spectrometer (Q TRAP), AB4500 Q TRAP UPLC/MS/MS System, equipped with an ESI Turbo Ion-Spray interface, operating in positive and negative ion mode and controlled by Analyst 1.6.3 software (AB Sciex) [32]. The ESI source operation parameters were as follows: ion source, turbo spray, source temperature 550 °C, ion spray voltage (IS) 5500 V (positive ion mode)/-4500 V (negative ion mode), ion source gas I (GSI), gas II (GSII), curtain gas (CUR) were set at 50, 60, and 25.0 psi, respectively; the collision activated dissociation (CAD) was high. Instrument tuning and mass calibration were performed with 10 and 100 μmol/L polypropylene glycol solutions in QQQ and LIT modes, respectively. QQQ scans were acquired as MRM experiments with collision gas (nitrogen) set to medium. DP and CE for individual MRM transitions were performed with further DP and CE optimization. A specific set of MRM transitions was monitored for each period according to the metabolites eluted within this period.

### Differential metabolite selection and KEGG enrichment analysis

Combined with previous research results, we screened the metabolites of *C*. *yanhusuo* bulbs by comparing the fragment pattern, retention time and *m/z* value of metabolites with those in a self-built database (MetWare, Wuhan, China). Partial least squares discriminant analysis (PLS-DA) was used to maximize the metabolomic differences between samples. The relative importance of each metabolite to the PLS-DA model was examined using variable importance in projection (VIP) [33]. Based on VIP≥1 and fold change≥1.5 or fold change ≤0.67, differential metabolites (DAMs) between groups were selected. First, the identified metabolites were annotated using the KEGG Compound database (http://www.kegg.jp/kegg/compound/) [34]. Second, successfully annotated metabolites were mapped into the KEGG pathway database (http://www.kegg.jp/kegg/pathway.html). Finally, the path of metabolites with significant regulation was input into MSEA (metabolite set enrichment analysis), and its significance was determined by the p value of the hypergeometric test.

### RNA extraction and transcriptome sequencing

RNA of each bulb sample was extracted by TRIzol reagent (Invitrogen) following the manufacturer’s protocol, and 1% agarose gel electrophoresis was used to detect whether RNA was degraded and contaminated. NanoPhotometer^®^ spectrophotometer (IMPLEN, CA, USA) and Qubit^®^ RNA Assay Kit in Qubit^®^2.0 Fluorometer (Life Technologies, CA, USA) were used to measure the purity and concentration, respectively. RNA integrity was checked using the RNA Nano 6000 Assay Kit of the Bioanalyzer 2100 system (Agilent Technologies, CA, USA). A total of 1 μg RNA from each sample was used as input material for RNA sample preparation. According to the manufacturer’s recommendations, the cDNA library was constructed using the NEBNext^®^ UltraTM RNALibrary Prep Kit for Illumina^®^ (NEB, USA), and the index codes were added to the attribute sequence of each sample. The clustering of the index coded samples was performed on a cBot Cluster Generation System using TruSeq PE Cluster Kit v3-cBot-HS (Illumina) according to the manufacturer’s instructions. After cluster generation, the library preparations were sequenced on an Illumina HiSeq platform, and 125 bp/150 bp paired end reads were generated. Clean data were obtained by filtering the raw data using fastp [35]. It is mainly used for deleting reads with adapters, including poly-N and low-quality (Q≤20) reads from raw reads. In addition, the Q20, Q30, GC content and sequence duplication level of the clean data were calculated. All subsequent analyses were based on clean reads. Transcriptome sequence assembly was performed using Trinity (v2.11.0) [36]. Related transcripts were reorganized into "gene" clusters by Corset [37] (https://github.com/trinityrnaseq/trinityrnaseq). Based on the following databases, the gene functions were annotated using diamond or Hmmer, NR (NCBI nonredundant protein sequence), SwissPort (manually annotated and reviewed protein sequence database), TrEMBL (a variety of new documentation files and the creation of TrEMBL, a computer annotated supplement to SWISS-PROT), KEGG (Kyoto Encyclopedia of genes and genomes), GO (gene ontology), KOG/COG (COG: protein homology cluster, KOG: eukaryotic homology group), and Pfam (Protein family).

### Differential expression analysis

The transcripts successfully assembled and spliced by Trinity [36] were used as reference sequences (Ref), the clean reads of each sample were mapped back to this ref in RSEM (v1.2.15) [38], and the bowtie2 [39] mismatch was set to the default value of 0. The relative expression level of a single gene was indicated by FPKM (expected number of Fragments Per Kilobase of transcript sequence per Millions of base pairs sequenced), which was the result of normalization of the read count of each sample. Differential expression analysis between different groups was performed using the DESeq2 software package (v1.6.3) [40, 41]. The p-value was adjusted using the method of Benjamin and Hochberg to control the false discovery rate. Genes with an adjusted p-value (Padj) < 0.05 and | log _2_ fold change |≥1 were designated differentially expressed genes (DEGs).

### High-performance liquid chromatographic (HPLC) analysis

First, the bulb samples of *C*. *yanhusuo* were crushed and dried. Then, 0.5 g of each sample powder was precisely weighed and put into a triangular flask with a stopper, dissolved in 20 ml of 50% methanol solution, extracted by ultrasound at room temperature for 20 min, and then cooled. Microporous membranes (0.22 μm) were used to filter the supernatant to obtain the test sample solution. Second, the standard references of protoopioid, palmatine, dehydrocorydrine, corydine, papaverine, tetrahydrojatrorrhizine, tetrahydropalmatine, tetrahydroberberine and corydaline were accurately weighed in appropriate amounts, a small amount of methanol was added, the samples were shaken well, and then the samples were ultrasonically dissolved to prepare mixed reference solutions, which were stored at 4 °C for standby.

HPLC conditions: Waters XBridge chromatographic column (4.6×250 mm, 5 μm); mobile phase A was 0.1% phosphoric acid solution (adjusted by triethylamine to pH 6.02), gradient elution (0~5 min, 90% A; 5~15 min, 90%A~80% A; 15~35 min, 80% A; 35~65 min, 80% A~45% A; 65~75 min, 45%A~5% A; 75~80 min, 5% A; 80~85 min, 5% A~90% A), detection wave length 280 nm, flow rate 1.0 mL/min, injection volume 10 μL. The column temperature was 30 °C.

### Sequence and data accession numbers

The whole set of transcript data can be found in the National Center for Biotechnology Information (NCBI) SRA database PRJNA907183. Widely targeted metabolome raw data and metabolites can be found in the metabolome database (https://www.metabolomicsworkbench.org/), and the DOI for this project (PR001845) is: http://dx.doi.org/10.21228/M87B0HPR001845.

### Data statistics and analysis

The experimental data was analyzed by IBM SPSS 26 and charted by Origin 2018.

## Results

### Widely targeted metabolome analysis at different developmental stages and parts of *Corydalis yanhusuo* bulb

**Fig 2:**
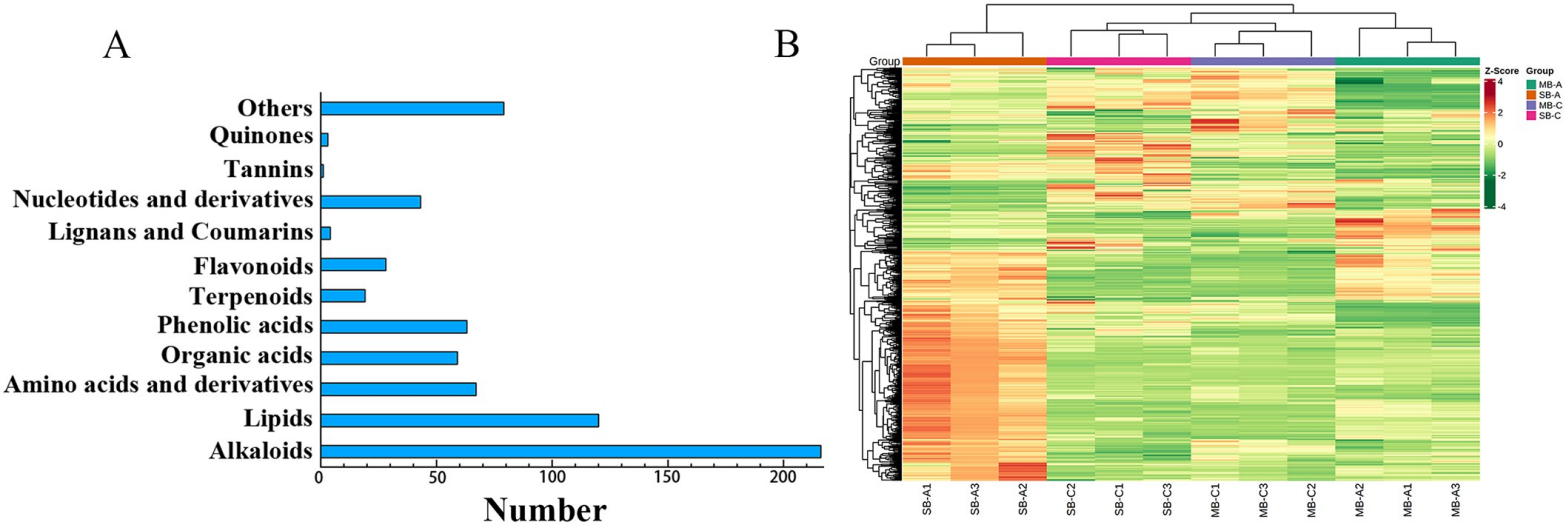
Number and heat-map of metabolite profiles of samples. (A) Number of different types of metabolites in all samples. (B) Clustering heat-map of the metabolites detect in the total samples. Each sample is visualized in a column and each metabolite is represented by a single row. Red indicates high abundance, whereas low relative metabolites are shown in green. MB-A: mother-bulb expansion period, SB-A: son-bulb expansion period, MB-C: mother-bulb maturity period, SB-C: son-bulb maturity period.

Widely targeted metabolome sequencing of *C*. *yanhusuo* bulbs was performed by UPLC-ESI MS/MS system [42], and its metabolites were successfully identified and annotated in self-built database (the MetWare database). A total of 702 metabolites were identified in all samples (Fig 2A), including 216 alkaloids, 120 lipids, 67 amino acids and their derivatives, 59 organic acids, 63 phenolic acids, 19 terpenoids, 28 flavonoids, 4 lignin and coumarins, 43 nucleotides and their derivatives, 1 tannin, 3 quinones and 79 other substances. In the clustering heatmap, we observed that all biological replicates cluster together (bottom of the figure), which indicated that the metabolomic data were highly reproducible (Fig 2B). Therelative abundance of metabolites accumulated in different developmental stages and parts showed obvious separation among experimental groups, indicating that those are obvious differences in the metabolic characteristics of them. As shown in Fig 2A, the secondary metabolites detected in bulbs included alkaloids, phenolic acids and flavonoids, and alkaloids accounted for 30.7% of the total metabolites, indicating that the secondary metabolites mainly focus on the synthesis of alkaloids in *C*. *yanhusuo* bulbs [3].

### Comparison of metabolites produced by the four groups of samples

**Fig 3:**
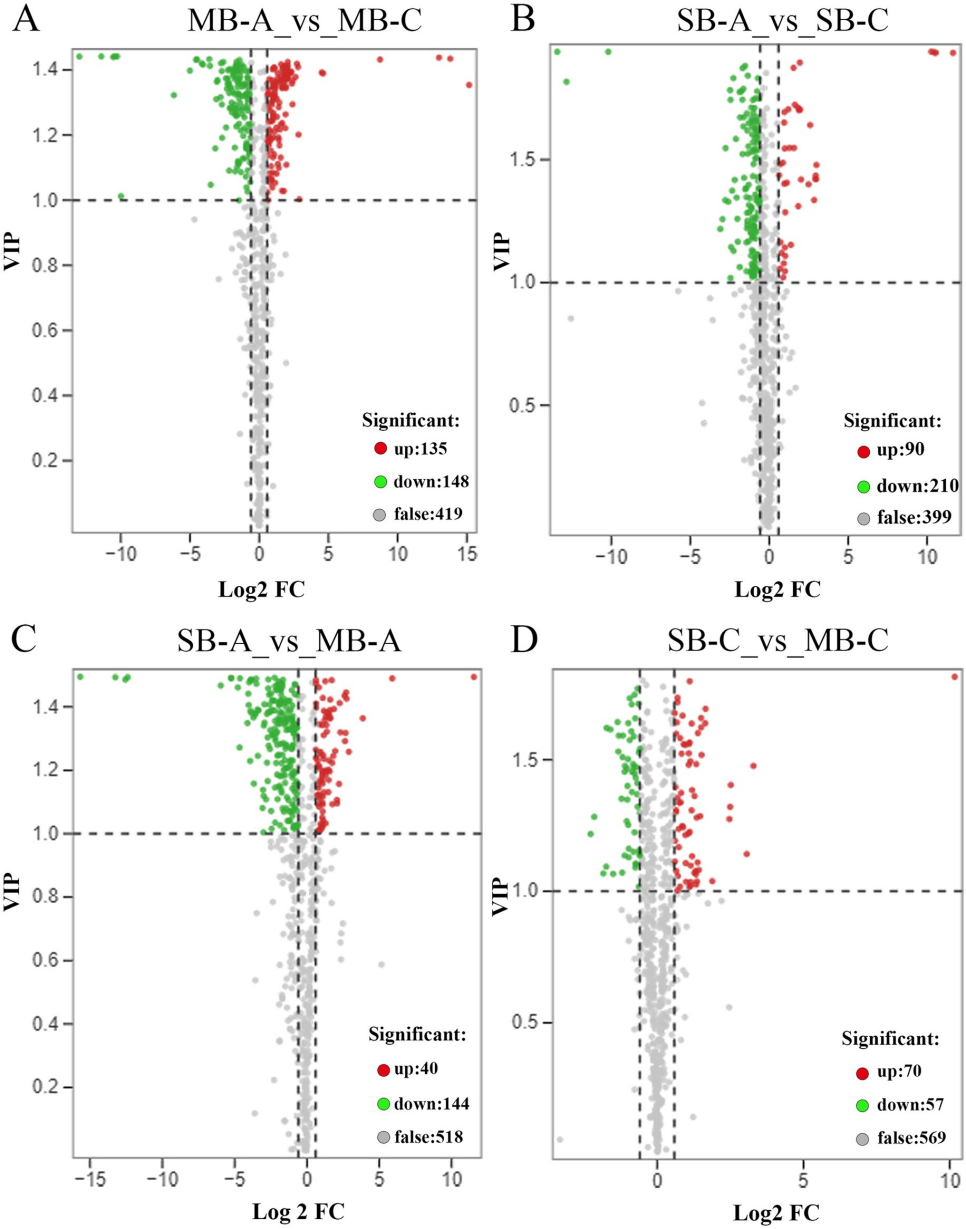
Volcano plot of differential metabolites between(A) MB-A vs MB-C, (B)SB-A vs SB-C, (C)SB-A vs MB-A and (D)SB-C vs MB-C; MB-A: mother-bulb expansion period, SB-A: son-bulb expansion period, MB-C: mother-bulb maturity period, SB-C: son-bulb maturity period. Each point in the volcano map correspond to a detected metabolite, in which the abscissa represent the logarithm of the fold change of metabolite abundance in the two groups of samples, and the ordinate represent the variable importance value (VIP) in the project. The red dots indicate the up-regulated DAMs, the green dots indicate the down-regulated DAMs, and the gray dot indicates the detected metabolite with no significant difference.

**Fig 4:**
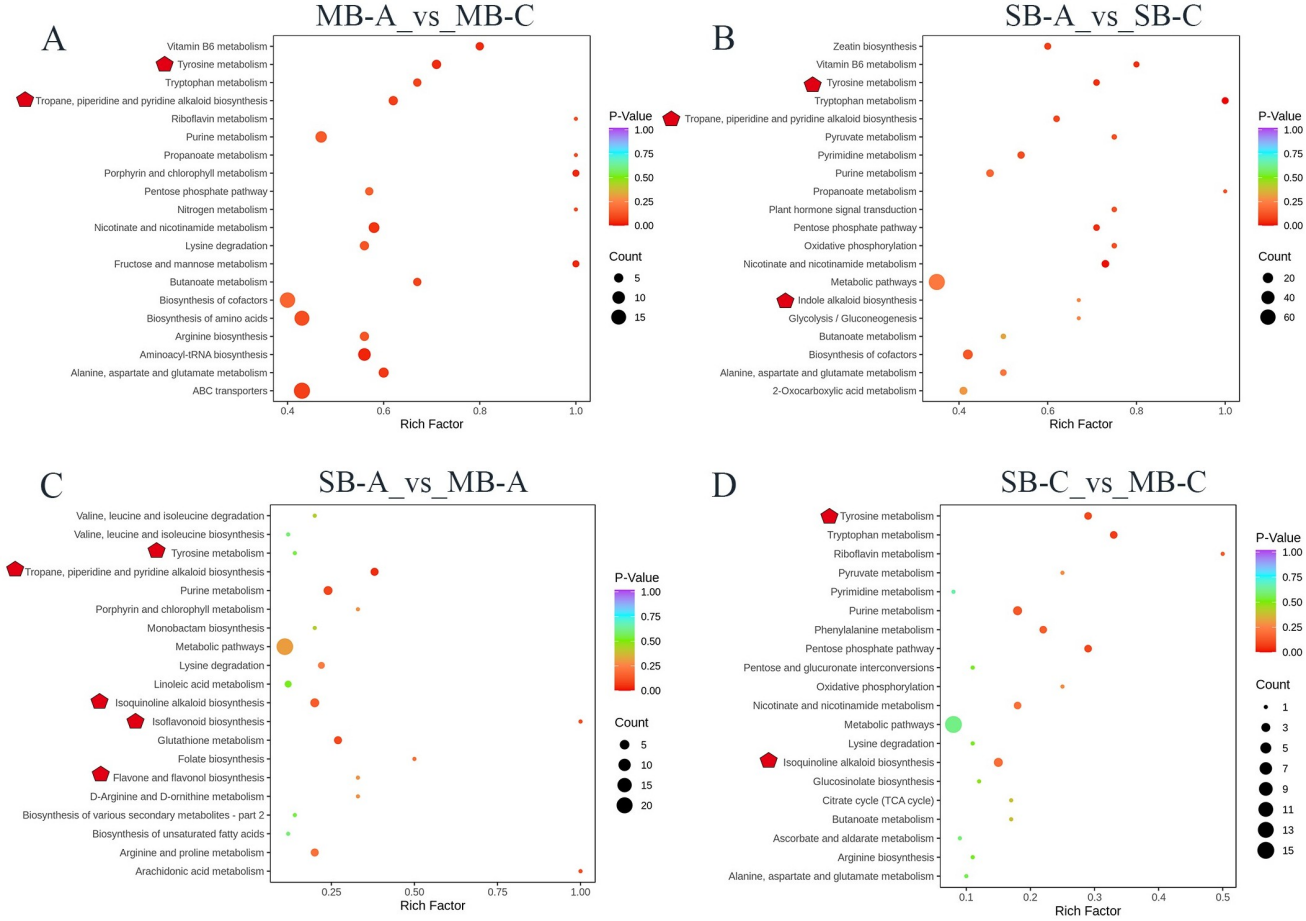
Top 20 KEGG pathway enrichment of DAMs between (A) MB-A vs MB-C, (B) SB-A vs SB-C, (C)SB-A vs MB-A and (D) SB-C vs MB-C. MB-A: mother-bulb expansion period, SB-A: son-bulb expansion period, MB-C: mother-bulb maturity period, SB-C: son-bulb maturity period. The dot color represents the q-value, and the dot size represented the number of differential expressed genes. Red stars indicate metabolic pathways related to secondary metabolite in *C*.*yanhusuo*.

To explore the dynamic changes in metabolite accumulation in different developmental stages and suborgans, we compared four samples in pairs. Based on the criterion of a variable importance in projection (VIP) ≥ 1 and a fold change ≥1.5 or ≤0.67, the differentially accumulated metabolites (DAMs) between pairs of samples (MB-A vs MB-C, SB-A vs SB-C, SB-A vs MB-A and SB-C vs MB-C) were selected (Fig 3). The numbers of up-accumulated and down-accumulated metabolites in MB-A vs MB-C and SB-A vs SB-C were 135 and 148, 90 and 210, respectively (Fig 3A and 3B). There were 184 kinds of DAMs between SB-A and MB-A (including 144 down-accumulated and 40 up-accumulated compounds in the MB-A samples) (Fig 3C) and 127 kinds of DAMs between SB-C and MB-C (including 57 down-accumulated and 40 up-accumulated compounds in the MB-C samples) (Fig 3D). To explore the function of these DAMs, we performed KEGG pathway enrichment analysis. Enrichment of the top 20 pathways showed that DAMs were mainly enriched in the metabolic pathways of primary products and secondary products, such as the metabolism pathways of amino acids, nucleotides, alkaloids and flavonoids. Tyrosine metabolism was significantly enriched in the four comparison groups, which may be because tyrosine was used as a reaction substrate to synthesize all kinds of downstream BIAs. Excluding SB-C vs MB-C, the pathways of tropine, piperidine and pyridine alkaloid biosynthesis were significantly enriched in the other three groups (Fig 4A–4C). The pathway of indole alkaloid biosynthesis was only enriched in SB-A vs SB-C (Fig 4B), and the pathway of isoquinoline alkaloid biosynthesis was significantly enriched in SB-A vs MB-A, SB-C vs MB-C (Fig 4C and 4D). In SB-A vs MB-A group, isoflavonoid biosynthesis and flavonoid and flavonol biosynthesis were also significantly enriched (Fig 4C). KEGG pathway enrichment demonstrated that the accumulation patterns of metabolites were different in the different developmental stages and suboranges of *C*. *yanhusuo* bulbs.

### Overview and location of benzylisoquinoline alkaloids (BIAs) in *C*. *yanhusuo* bulbs

Among 702 metabolites, 216 alkaloids were identified, accounting for 30.8% of the total metabolites. A total of 111 of 216 were identified as benzylisoquinoline alkaloids (BIAs). Protoberberine alkaloids, for which most compounds take berberine as the skeleton, include (S)-canadine [43], tetrahydropalmatine (THP) [44], scoulerine [45], corydaline [44], jatrorrhizine [46], tetrahydrocolumbamine [47], cheilanthifoline [48] and other metabolites (S1 Table). Aporphine alkaloids with glaucine as the skeleton include d-glaucine [49], magnoflorine [50], corydine [51], nantenine [51], thaliporphine [52], (S)-lirioferine [53], isocorydine [54] and others (S1 Table). Protoberberine-type and aporphine-type alkaloids have been -showed to be the most abundant alkaloids in *C*. *yanhusuo* bulbs [14]. Few opiate-type alkaloids were found (S1 Table), such as protopine [51], pseudoprotopine [55] and allocryptopine [51]. Additionally, other alkaloids were also found in our study, including 43 phenolic amines, 10 indole alkaloids, 1 sesquiterpene alkaloid, 2 pyridine alkaloids, 3 pyrrole alkaloids, 1 quinorisidine alkaloid, 1 tropan alkaloid, 2 piperidine alkaloids, 32 alkaloids, and 2 terpenoid alkaloids. These results fully demonstrate that alkaloids are the major secondary metabolites in *C*. *yanhusuo* bulbs, especially BIAs, providing a theoretical basis for the identification of key compounds in the BIA synthesis pathway.

BIAs-producing plants are mainly concentrated in Papaveraceae, Ranunculaceae, Berberidaceae, Menispermaceae and other plant groups, such as *Opium poppy* [22], *Coptis japonicus* [56] and *Macleaya cordata* [57]. The existing research results showed that BIAs biosynthesis and accumulation varied with species, tissues and organs. Morphine, noscapine, papaverine and other end-products or intermediates are significantly accumulated in latex, which is a kind of cytoplasm produced by specialized laticifers found in all *Opium poppy* organs. However, sanguinarine, most proberberine, protopine and some intermediates of the benzo[c]phenanthridine pathway accumulate significantly in roots, indicating that these substances probably accumulate in cells rather than laticifers [58, 59]. Moreover, the accumulation of proberberine is not limited to the endoderm of roots but occurs more widely in the cortex of rhizomes [60]. At present, BIAs have been confirmed to be the most important chemical substance in *C*. *yanhusuo*, and their content in tubers is significantly higher than that in leaves, showing obvious tissue specificity [21]. The temporal and spatial accumulation of BIAs may be related to the expression pattern of biosynthetic genes. Therefore, the transcripts were useful sources for gene expression analysis, which helped in the further identification of unigenes and pathways of biosynthesis.

### Transcriptome characteristics of different parts and different development stages of *Corydalis yanhusuo* bulb

To identify the genes involved in BIAs synthesis in *C*. *yanhusuo*, samples from the expansion period of mother-bulb (MB-A), the expansion period of son-bulb (SB-A), the maturity period of mother-bulb (MB-C), and the maturity period of son-bulb (SB-C) were sequenced by the Illumina HiSeq 2000 platform. A total of 1054 million clean reads were obtained, including 158.12 GB of clean bases, and the base percentage of Q30 was above 92% in all samples (S2 Table). After assembling and splicing, a total of 190269 transcripts were produced, with an average length of 891 bp, of which the lengths of N50 and N90 were 1431 bp and 361 bp, respectively. After hierarchical clustering, 183631 unigenes were identified, accounting for 99% of transcripts, with an average length of 914 bp, among which 32820 unigenes were over 1000 bp and 19561 unigenes were over 2000 bp in length (S3 Table). To analyse the functions of the unigenes obtained, the genes were annotated by seven nucleotide and protein databases (KEGG, NR, SwissProt, Trembl, KOG, GO, Pfam). In this study, 60126 unigenes were annotated in the KEGG database (32.74%), 82253 in the NR database (44.79%), 55720 in the SwissProt database (30.34%), 81579 in the Trembl database (44.43%), 49577 in the KOG database (27.00%), 68079 in the GO database (37.07%), and 53700 in the Pfam database (29.24%). Among the 183631 unigenes, 85812 were annotated in at least one database, accounting for 46.73% of the total unigenes (Table 1).

**Table 1:** Annotation distribution of unigenes in the database.

Database	Number of unigenes	Percentage (%)
KEGG	60126	32.74
NR	82253	44.79
SwissProt	55720	30.34
Trembl	81579	44.43
KOG	49577	27
GO	68079	37.07
Pfam	53700	29.24
Annotated in at least one Database	85812	46.73
Total Unigenes	183631	100

### Differentially expressed genes (DEGs) in *C*. *yanhusuo* bulbs

**Fig 5:**
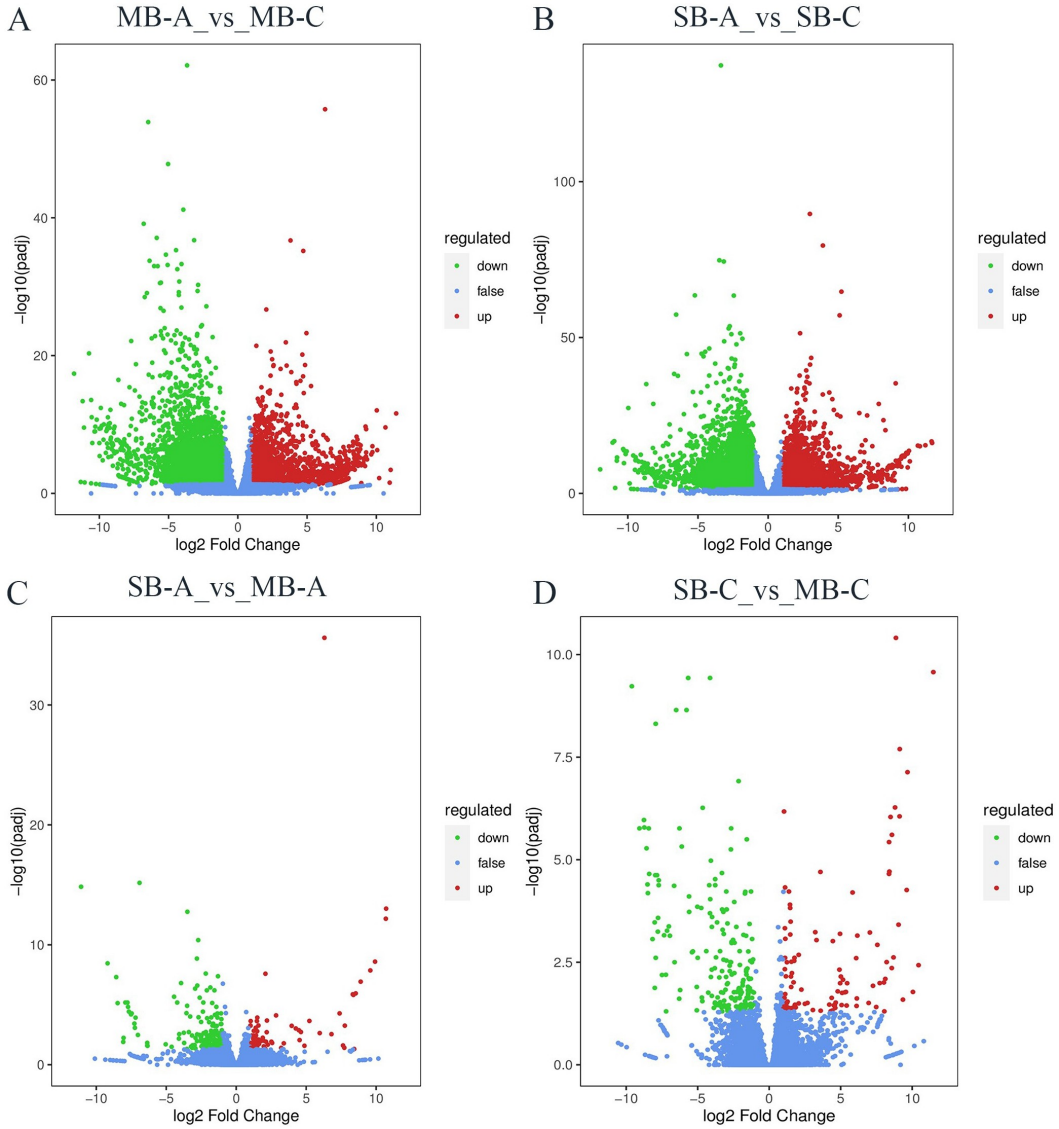
Volcano plot of Differential expression of genes between *C*. *yanhusuo* bulb between (A) MB-A vs MB-C, (B) SB-A vs SB-C, (C)SB-A vs MB-A and (D) SB-C vs MB-C; MB-A: mother-bulb expansion period, SB-A: son-bulb expansion period, MB-C: mother-bulb maturity period, SB-C: son-bulb maturity period. Each point in the volcano map correspond to a detected gene, in which the abscissa represent the logarithm of the fold change of gene expression in the two groups of samples, and the ordinate represent the significance level in the project. The red dots indicate the up-regulated differential gene, the green dots indicate the down regulated differential gene, and the blue dots indicate genes without significant changes.

To analyse the dynamic changes at the gene expression level during the development of *C*. *yanhusuo* bulbs, the abundance changes of pairwise samples were evaluated by converting read count to fragments per kilobase per million (FPKM). The differentially expressed genes (DEGs) were filtered with a threshold of padj <0.05 and log_2_ fold change≥1. Volcano plots were used to show the relationship between P values and log _2_ fold change (Fig 5). A total of 7370 differentially expressed genes (DEGs) were detected between MB-A and MB-C, including 3063 upregulated DEGs and 4307 downregulated DEGs in MB-C (Fig 5A). Compared with SB-A, 12332 DEGs were found in SB-C samples, of which 5734 genes were upregulated and 6598 genes were downregulated (Fig 5B). A total of 227 DEGs were found between SB-A and MB-A samples, including 80 upregulated genes and 147 downregulated genes in MB-A (Fig 5C). Compared with SB-C, a total of 314 DEGs were obtained in MB-C, including 104 upregulated genes and 210 downregulated genes (Fig 5D). During the whole development of *C*. *yanhusuo* bulbs, whether mother-bulb or son-bulb, the number of DEGs changed significantly between the expansion period and maturity period (7307 DEGs and 12332 DEGs, respectively), while at the same development stage (expansion stage or maturity stage), the number of DEGs between two suborgan parts (mother-bulb or son-bulb) was lower (227 DEGs and 314 DEGs, respectively). This result indicates that most transcripts were significantly regulated at different developmental stages of the bulb but were relatively stable between the mother bulb and son bulb. This may be related to the fact that both of them are classified as tubers, although there are differences in their occurrence positions. The gene expression of bulbs was relatively active at the expansion stage, so the synthesis and accumulation of metabolites could be rapidly achieved. For the maturity stage, bulb development has reached maximum maturity, and the expression level of genes is relatively stable.

### KEGG pathway enrichment of DEGs in *C*. *yanhusuo* bulb development

**Fig 6:**
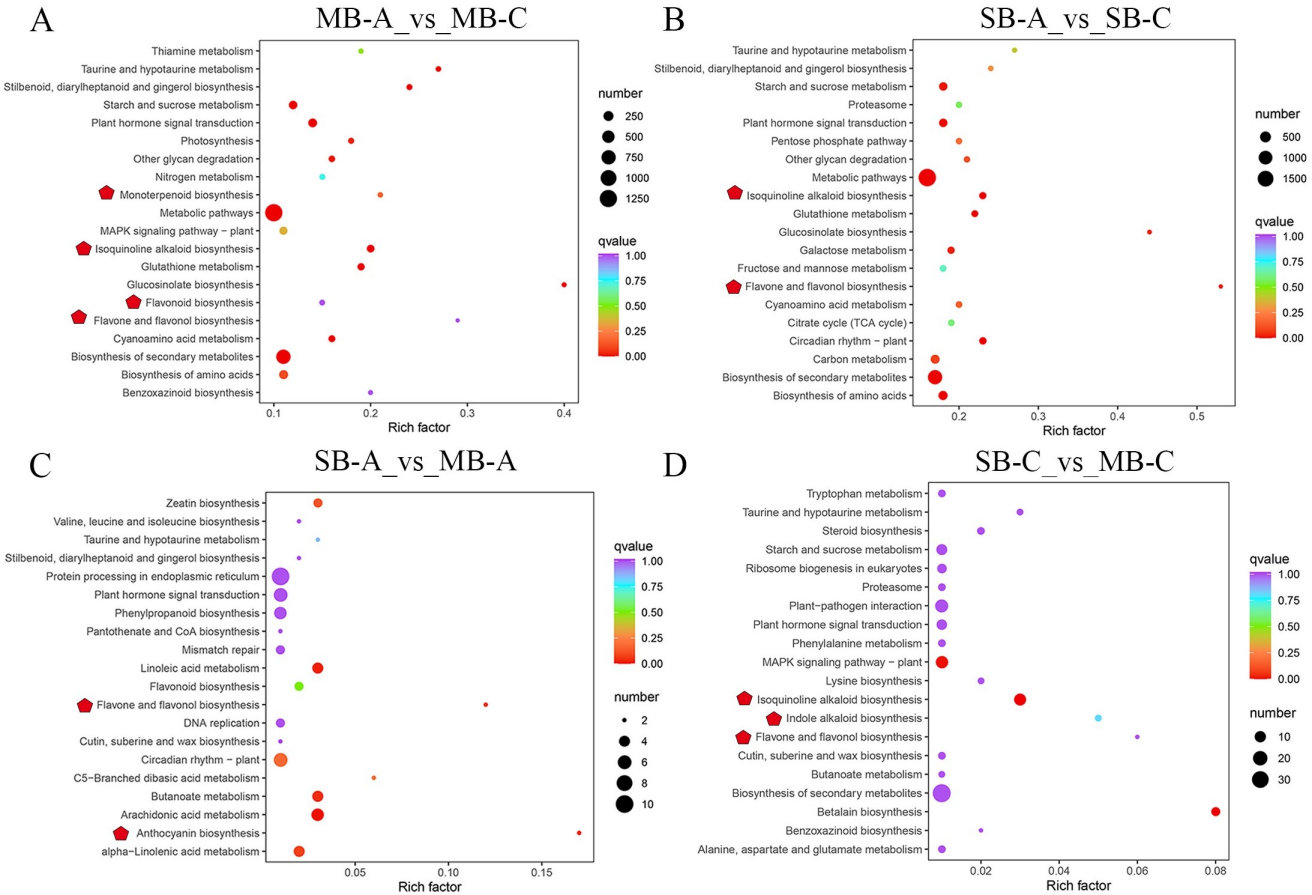
Top 20 KEGG pathway enrichment of DEGs between (A) MB-A vs MB-C, (B) SB-A vs SB-C, (C)SB-A vs MB-A and (D) SB-C vs MB-C. MB-A: mother-bulb expansion period, SB-A: son-bulb expansion period, MB-C: mother-bulb maturity period, SB-C: son-bulb maturity period. The dots colored represent the q-value, and the dot size represents the number of differential expressed genes. Red stars indicate metabolic pathways related to secondary metabolite synthesis in *C*.*yanhusuo*.

In this study, KEGG analysis found that 6701 unigenes were related to secondary metabolic synthesis, of which 457 unigenes were annotated to participate in the synthesis of isoquinoline alkaloids, followed by tropine, piperidine and pyridine alkaloid biosynthesis (233 unigenes) and indole alkaloid biosynthesis (40 unigenes) (S4 Table). We selected the top 20 pathways with the most significant enrichment of DEGs for display with a scatter plot (Fig 6). KEGG pathway enrichment showed that these DEGs were mainly enriched in signal transduction and secondary metabolite synthesis pathways. In the pairwise comparison of the four samples, the pathways of flavonoids and flavonols were significantly enriched, an important secondary metabolite pathway of plant pigments. Excluding SB-A vs MB-A, the isoquinoline alkaloid biosynthesis pathway was significantly enriched in the other three groups, and the enrichment factors in MB-A vs MB-C and SB-A vs SB-C were 0.20 and 0.23, respectively (S5 Table), which revealed that isoquinoline alkaloids were important secondary metabolites in *C*. *yanhusuo* bulbs (Fig 6A, 6B and 6D). MB-A vs MB-C -also concentrated on the pathway of monoterpene biosynthesis and flavonoid biosynthesis (Fig 6A). The anthocyanin biosynthesis pathway and indole alkaloid biosynthesis were enriched in SB-A vs MB-A and SB-C vs MB-C, respectively (Fig 6C and 6D). This conclusion preliminarily confirmed the obvious differences in gene expression in the two developmental stages and two suborgans of the bulb.

### Common pathways for biosynthesis of various BIAs and screening of candidate genes for *C*. *yanhusuo* bulb

**Fig 7:**
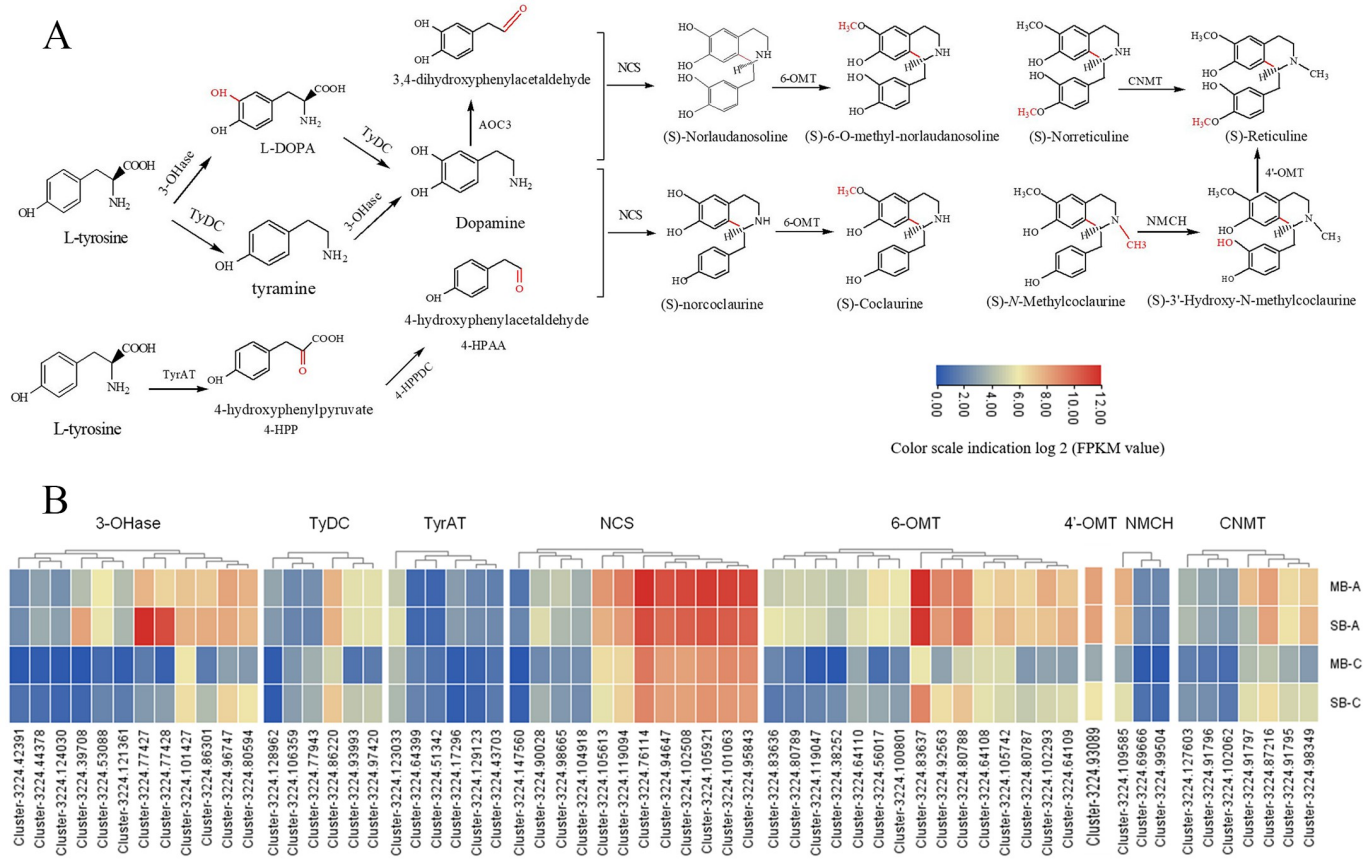
Common pathway and candidate genes for BIAs synthesis of *C*.*yanhusuo* bulb. A: Common pathway of synthesis of BIAs. B: The FPKM value of multiple transcripts of candidate genes involve in the common pathway. Tyrosine 3-hydroxylase (3-OHase), tyrosine decarboxylase (TyDC), tyrosine aminotransferase (TyrAT), norcoclaurine synthese (NCS), (S)-3′-hydroxy-N-methylcoclaurine-4′-O-methyltransferase (4′- OMT), (S)-norcoclaurine 6-O-methyltransferase (6-OMT), (S)–coclaurine-N-methyltransferase (CNMT), (S)-N-methylcoclaurine-3′-hydroxylase (NMCH).

BIAs biosynthesis shares common steps among different plant species, especially in the upstream pathway of synthesis of (S)-reticuline [31, 56]. Combined with the previous results, we constructed a common pathway for the synthesis of various BIAs and screened out candidate genes (Fig 7). A series of BIAs synthesis practically came from tyrosine. Tyrosine is catalysed by various functional enzymes from different protein families, including cytochrome P450, FAD-linked oxidoreductase, 2-oxoglutarate/Fe(II)-dependent dioxygenase, SAM-dependent O-methyltransferases (OMTs) and N-methyltransferases (NMTs), to yield (S)-reticuline, an important precursor intermediate for the formation of downstream BIAs. We observed and analysed the abundance of 62 transcripts encoding eight functional enzyme genes in *C*. *yanhusuo* bulbs, including *3-OHase*, *TyDC*, *TyrAT*, *NCS*, *6-OMT*, *4′-OMT*, *CNMT* and *NMCH*, which participated in the common pathway (Fig 7A). The results were consistent with previous reports in *Opium poppy* [31] and *Coptis japonicus* [56], indicating that the common pathway of BIAs was relatively conserved. As shown in Fig 7B, most of the key enzymes were encoded by multiple single genes. Twelve unigenes were predicted to encode 3-OHase, which was the rate-limiting enzyme in the first step of L-dopamine synthesis. Fifteen unigenes were predicted to encode 6-OMT, a rate-limiting key enzyme for BIAs production. Only one unigene was predicted to encode 4′-OMT, which is involved in the last step for the formation of (S)-reticuline, a key branch-point intermediate for the synthesis of BIAs. There were six unigenes predicted to encode TyDC and TyrAT, which are involved in the synthesis of dopamine and 4-hydroxyphenylpyruvic acid, respectively. NCS catalysed the condensation reaction of dopamine and 4-HPAA, which was the first step in the biosynthesis of BIAs and was encoded by 10 single unigenes. CNMT and NMCH jointly participated in the synthesis of (S)-3’-hydroxy-N-methylcoclaurine and were encoded by seven and three unigenes, respectively. For both the mother and son bulbs, the expression level of most unigenes in the expansion stage (MB-A and SB-A) was higher than that in the maturity stage (MB-C and SB-C). In the same period, the expression level of most unigenes was not significantly different between mother-bulb and son-bulb, yet a few transcripts of *3-OHase*, *TyDC*, *NCS*, *6-OMT*, *4′-OMT*, *CNMT* and *NMCH* showed significant differences. These enzymes show a fuzzy regulation pattern between the mother-bulb and son-bulb, which may be due to the lack of strict regulations of these functional enzyme reactions or some enzymes having their own distribution in the suborgans.

### Construction of biosynthetic pathways of various BIAs and screening of candidate genes for *C*. *yanhusuo* bulb

**Fig 8:**
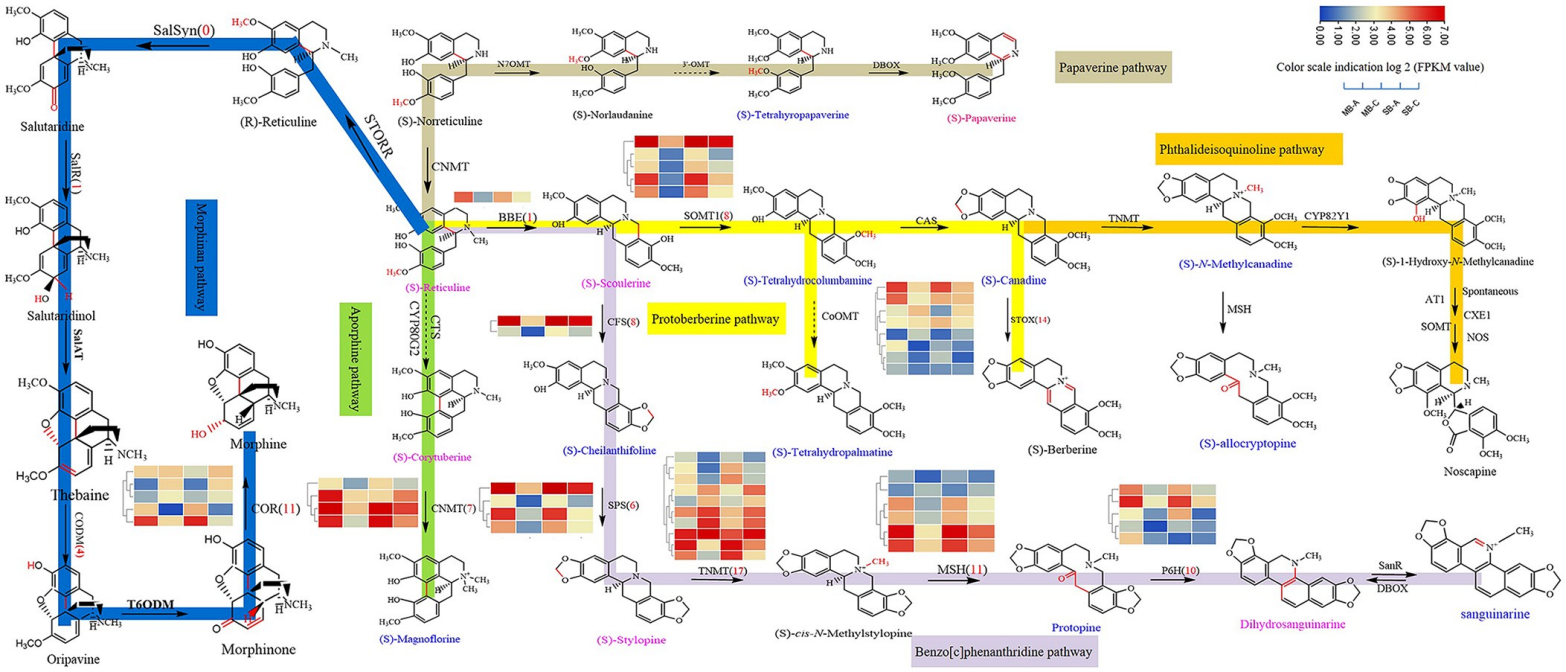
Summary of BIAs pathway and screening of candidate genes of *C*. *yanhusuo* bulb. Heatmap of multiple transcripts by the FPKM values matching each gene is shown side by side near the gene names. The red number in the bracket represents the number of this functional enzyme. Norreticuline 7-O-methyltransferase (N7OMT), berberine bridge enzyme (BBE), scoulerine 9-O-methyltransferase (SOMT), canadine synthase (CAS), stylopine synthase (SPS), (S)-tetrahydroxyprotoberberine oxidase (STOX), columbamine O-methyltransferase (CoOMT), cheilanthifoline synthase (CFS), tetrahydroprotoberberine-N-methyltransferase (TNMT), methylstylopine hydroxylase (MSH), dihydrobenzophenanthridine oxidase (DBOX), protopine-6-hydroxylase (P6H), 1,2-dehydroreticuline synthase/reductase (STORR), salutaridine synthase (SalSyn), salutaridine reductase (SalR), salutaridinol-7-Oacetyltransferase (SalAT), thebaine-6-O-demethylase (T6ODM), codeinone reductase (COR), codeine-O-demethylase (CODM), noscapine synthase (NOS), (13S,14R)-1,13-dihydroxy-Nmethylcandine 13-O-acetyltransferase (AT1), 3-O-acetylpapaveroxine carboxylesterase (CXE1).

At present, the alkaloids reported in *C*. *yanhusuo* are mainly benzylisoquinoline alkaloids (BIAs), and the pathway and enzymes involved in BIAs biosynthesis are relatively clear but not complete. In our study, based on the UPLC–MS/MS platform and self-built database, most metabolites of BIAs were detected in *C*. *yanhusuo* bulbs (Fig 8 and S1 Fig). The blue labelled substances were nondifferentially accumulated metabolites (non-DAMs) in the metabolic pathway, while those marked in pink were differentially accumulated metabolites (DAMs) (Fig 8 and S1 Table). However, we did not find metabolites related to the phthalideisoquinoline pathway (orange) and the morphine pathway (blue) in bulbs, and this result was consistent with a previous study [21]. However, (S)-allocryptopine, as the starting material for the noscapine pathway, was detected in our samples, which may be related to the structural analogue of protopine. Most of the metabolites in the protoberberine pathway (yellow), aporphine pathway (green) and benzo[C]phenanthridine pathway (purple) were discovered. Although berberine was not found, similar substances such as epiberberine, berberastine and oxyepiberberine were detected in metabolites (S1 Table). Epiberberine and berberine are isomers, and berberastine and oxyepiberberine may be formed by methylation or hydroxylation of berberine. This also further proved that protoberberine and protoopioid alkaloids were the main compounds in *C*. *yanhusuo* bulbs.

According to the tblastn results of transcriptome data, we found almost all homologues of enzymes involved in the synthesis pathway of BIAs (Fig 8). A total of 92 nonredundant unigenes were obtained. Most unigenes were annotated to participate in the protoberberine and benzo[C]phenanthridine pathways, such as *BBE*, *CNMT*, *CFS*, *SPS*, *SOMT1*, *STOX*, *MSH*, *TNMT* and *P6H*, which was consistent with the detected metabolites, indicating that gene expression was synchronized with the biosynthesis of protoberberine and protopine alkaloids. However, few functional enzyme homologues involved in the morphinan pathway (blue) were found in the tblastn results. Meanwhile, no morphine, codeine or other substances were detected in our metabolites. Strangely, we found tetrahydropalmatine in the metabolites but did not find the corresponding functional enzymes (CoOMT), which were successfully cloned in *Opium poppy* (S6 Table), showing that the synthesis of tetrahydropalmatine may be regulated by other functional genes and not completely in line with that in *Opium poppy*, which needs further research and verification.

To explore the expression pattern of the screened candidate genes, heatmaps of multiple transcripts of functional enzymes in samples MB-A, MB-C, SB-A and SB-C are shown side by side near the gene names (Fig 8). The results showed that the expression levels of most single genes (*BBE*, *CFS*, *SPS*, *SOMT1*, *MSH*, *CNMT*, *TNMT*, *STOX* and *P6H*) were more active in the expansion stage of the bulb and were evidently higher in the expansion stage (MB-A and SB-A) than in the maturity stage (MB-C and SB-C). These significantly expressed genes were distributed in twelve gene families that synthesized downstream BIAs, indicating the rapid synthesis and accumulation of various BIA at the expansion stage of the bulb. However, the functional genes of *CNMT*, *TNMT*, *STOX* and *COR* showed fuzzy regulation patterns in the two developmental stages. This may be related to the fact that these enzymes were not strictly regulated. For the same developmental stage of the bulb, the expression level of most unigenes was not significantly different in the mother and son bulbs, indicating that the biosynthesis of BIAs was not related to the suborgans of the *C*. *yanhusuo* bulb, although the formation sites were different. However, some single genes of *CFS*, *SOMT1*, *CNMT* and *SPS* exhibited higher expression levels in SB-C than in MB-C, which indicated that these enzymes possibly had their own suborgan distribution. In general, the expression pattern of functional genes proved the following hypothesis: i, *C*. *yanhusuo* propagated asexually with bulbs as seeds, and although there were differences in their occurrence positions, the variation between the two suborgans was low, and perhaps there was no gene separation in essence, leading to little difference in gene expression levels. ii, the gene expression level was upregulated in the expansion stage of the bulb to achieve BIAs accumulation. iii, at the maturity stage of the bulb, BIAs accumulation has been nearly completed, accompanied by cell senescence and death, resulting in relatively low expression levels of functional genes.

### High-performance liquid chromatographic (HPLC) analysis of nine compounds of BIAs

**Fig 9:**
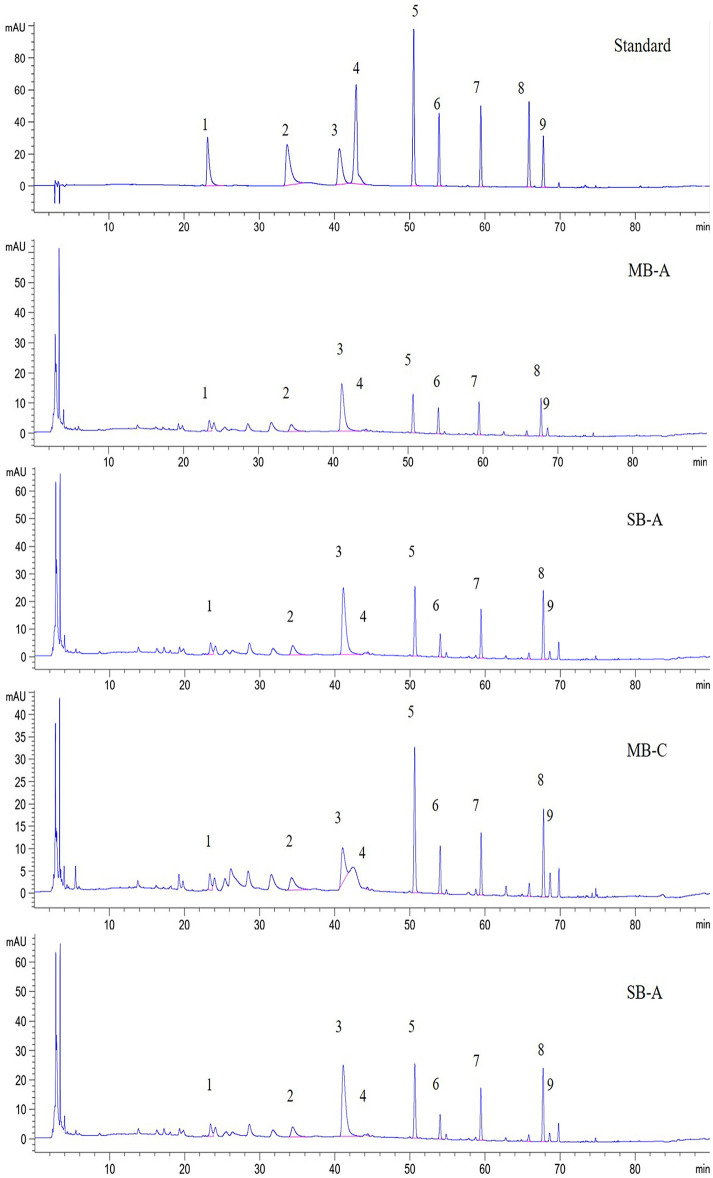
High performance liquid chromatographic analysis of nine compounds of BIAs at 280 nm. 1.protoopioid, 2.palmatine, 3. dehydrocorydrine, 4. corydine, 5. papaverine, 6.tetrahydrojatrorrhizine, 7. tetrahydropalmatine, 8. tetrahydroberberine, 9.corydaline.

To further verify the reliability of the differentially expressed transcriptome information in Fig 8, we selected nine compounds as BIAs representatives, namely, tetrahydropalmatine (THP), corydaline, protoopioid, dehydrocorydrine, tetrahydrojatrorrhizine, palmatine, papaverine, corydine and tetrahydroberberine, which were used as target compounds to standardize their content abundance in two developmental stages and suborgan parts of the bulb. We obtained the standard linear equations of nine standard samples by HPLC and then analysed the content of nine compounds in each sample (Fig 9 and S2 Fig). The results showed that there were differences in the contents of nine compounds at two developmental stages and in different suborgans (S2 Fig). The contents of all nine compounds were higher in the expansion stage (MB-A and SB-A) than in the maturity stage (MB-C and SB-C). At the same developmental stage, except for corydaline and tetrahydrojatrorrhizine, there was little difference in the content of seven compounds between mother and son-bulb, which was consistent with the previous transcriptome analysis to some extent (Fig 8). The content of corydaline in the SB-A sample was higher than that in MB-A, but tetrahydrojatrorrhizine showed the opposite result. Based on the transcriptional analysis and HPLC results, we confirmed the hypothesis that the accumulation of BIA was related to the expression pattern of related genes in bulbs.

### Phylogenetic analysis of candidate genes of O-methyltransferase for *C*. *yanhusuo* bulb

**Fig 10:**
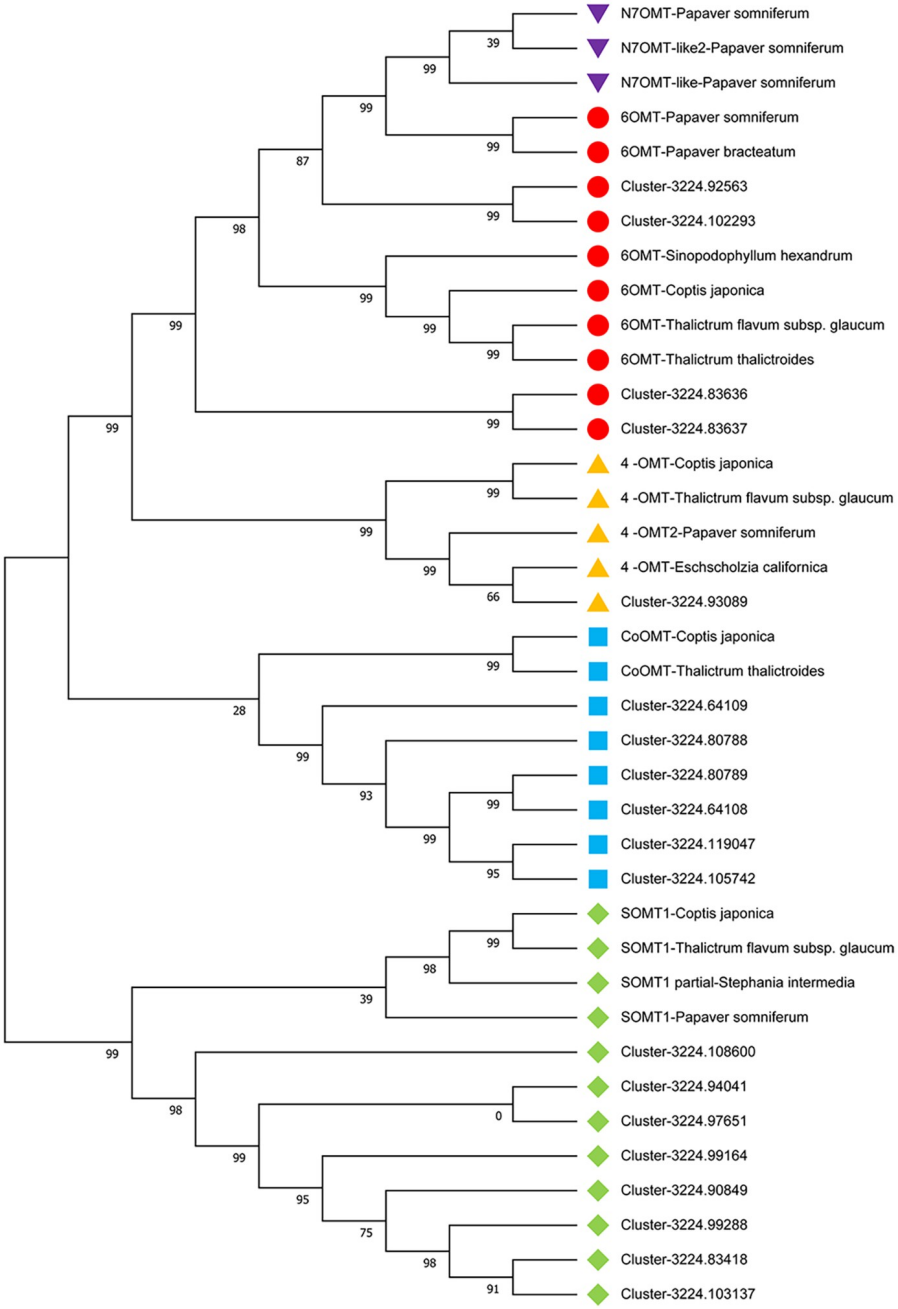
Phylogenetic analysis of OMT protein family in *C*. *yanhusuo* and other plant species. The phylogenetic tree showed that all OMT candidates formed five major clades: 6-OMT, 4′-OMT, N7OMT, CoOMT, and SOMT.

SAM-dependent O-methyltransferases (OMTs) are important functional enzymes in the BIA biosynthesis pathway of *C*. *yanhusuo* [21]. O-methyltransferase genes are highly homologous in sequence among different species, so phylogenetic analysis is able to classify the various OMTs. Tetrahydropalmatine (THP), an end product of O-methylated BIA, has analgesic and sedative medicinal value without addictive side effects [11]. Because it can be used as a substitute for opioids (highly addictive), it has attracted extensive attention, which is another important reason for our interest in OMT. Therefore, efforts to identify OMT (CoOMT) that catalyses the reaction from (S)-tetrahydrocolumbine to THP have been ongoing for many years. Here, we carried out phylogenetic analysis on OMT protein sequences of *C*. *yanhusuo* with other species, such as *Papaver somniferum*, *Coptis japonica*, *Sinopodophyllum hexandrum*, *Thalictrum flavum* subsp and *Macleaya cordata*, to provide some insights into the classification of the OMT protein family of *C*. *yanhusuo* (Fig 10). A phylogenetic diagram showed that all OMT candidates were clustered into five main branches: 6-OMT, 4-OMT, N7OMT, CoOMT and SOMT. Among them, N7OMT did not find the corresponding homologous enzyme in the transcripts of *C*. *yanhusuo*. Phylogenetic analysis found that four unigenes had high homology with 6-OMT of known reported species such as *Papaver somniferum*, *Coptis japonica* and *Thalictrum thalictroides*, indicating that the sequence of the Ps6-OMT protein was relatively conserved among different species. One single unigene was classified as 4-OMT, and six and eight unigenes were mapped to the CoOMT and SOMT branches, respectively. This result probably helped to explain the accumulation of THP in *C*. *yanhusuo* because it was reported that both CoOMT and SOMT could catalyse the reaction from tetrahydrocolumbine to THP [61]. Enzymes with CoOMT activity may appear in the translation products of 12 single genes in the CoOMT and SOMT branches (excluding two with insufficient length). S6 Table provides all sequence information used in the phylogenetic analysis. However, it is worth noting that six unigenes, which clustered as CoOMT, were annotated as 6-OMT in the tblastn results, indicating that their evolutionary branches were more closely related to CoOMT (*Coptis japonica* and *Thalictrum thalictroides*), so these six unigenes possibly played the role of CoOMT rather than 6-OMT in *C*. *yanhusuo* bulbs.

## Discussion

### Analysis of BIAs metabolites in *Corydalis yanhusuo* bulbs

The widely targeted metabolome showed that there were 702 metabolites in the *C*. *yanhusuo* bulbs. A total of 216 alkaloids were found, accounting for 30.7%, including 92 isoquinoline alkaloids and 25 aporphine alkaloids (S1 Table), indicating that BIAs were the main active components in *C*. *yanhusuo* bulbs [14]. The end-products of BIAs synthesis routes have been detected (Fig 8 and S1 Table), such as tetrahydropalmatine, tetrahydrocolumbamine, glaucine, dihydrosanguinarine, magnoflorine, papaverine, (S)-canadine, palmatine, corydaline and protopine. However, few compounds in the morpholane pathway (blue) and phthalideisoquinoline pathway (orange) were found. At present, these compounds have not been reported in Corydalis plants [21]. It is possible that the synthesis of morphine or noscapine required specific organs, such as laticifers [62, 63]. However, in *C*. *yanhusuo*, a Papaveraceae plant similar to *Opium poppy*, tissues and organs have undergone variation and differentiation during evolution (no laticifer), leading to an inability to synthesize morphine and noscapine. The accumulation of proberberine, sanguinarine and other substances has been indicated to occur in the rhizome (including the cortex) [59, 64]. It is worth noting that papaverine was detected in metabonomic analysis and HPLC results (S1 Table and S2 Fig), indicating that the synthesis region of papaverine is probably not limited to laticifers, so it can be synthesized in nonlaticifer plants, which requires more extensive discussion.

### Analysis of key enzyme (CoOMT) for tetrahydropalmatine synthesis

Tetrahydropalmatine (THP) is the main active compound in *C*. *yanhusuo* bulbs and has medicinal value in analgesia, sedation and nonaddiction [11]. Colombine O-methyltransferase (CoOMT) is a key enzyme involved in THP biosynthesis (Fig 8). However, combined with Nr annotation, no CoOMT homologues were found in the tblastn results. There were several reasons why we failed to detect homologues of genes in the transcriptome database: i, low expression made it difficult to detect; ii, the genes were specific in time and space and were not expressed in our sample tissues and stages; and iii, the sequence of protein varied between different species. We excluded the first two reasons because THP and palmatine were the second and fourth most abundant BIAs in bulbs, respectively [9], and metabonomic analysis and HPLC results successfully detected THP, which obviously accumulated, so the key genes responsible for their synthesis should be highly expressed and could be found. In addition, some results showed that there was no transport of BIAs between different organs in *C*. *yanhusuo*, BIAs were de novo synthesized in tubers or leaves [58], and accumulation of THP occurred throughout the whole growth process of bulbs [65]. Therefore, nondetection of CoOMT homologues in *C*. *yanhusuo* was likely due to protein sequence variation. Although CoOMT has been suggested to participate in palmatine biosynthesis in *Berberis wilsoniae* (Berberidaceae family) [66] and *Coptis chinensis* (Ranunculaceae family) [67, 68], the cDNA sequence encoding CoOMT was only cloned from *Coptis chinensis* cultured cells [10]. Among the OMTs related to BIAs synthesis, they were divided into four types, namely, PsSOMT2, CjCoOMT, 4’OMT and 6-OMT [61]. The phylogenetic analysis of all OMTs candidate sequences in the tblastn results of *C*. *yanhusuo* was carried out based on the reported OMT sequences of *opium poppy*, *Coptis chinensis* and other BIAs producing species. The results showed that (Fig 10), except for a single gene with an incomplete sequence, there were five complete unigenes that belonged to the same branch with CoOMT (*Coptis chinensis* and *Arabidopsis thaliana*), with high homology. Otherwise, it was reported that SOMT1 in *Opium poppy* could catalyse the transformation from tetrahydrocolumbine to THP [61]. We found six single unigenes of *C*. *yanhusuo* in the PsSOMT1 branch, excluding two single genes with incomplete sequences. Therefore, further research probably focused on cloning these single unigenes to verify whether their activity was related to THP synthesis at the protein level. There were nine single unigenes annotated with 6-OMT and one single gene annotated with 4’OMT (Fig 10). However, according to phylogenetic analysis results, no unigenes were found to be related to 3′-O-methyltransferase, which was predicted to be involved in the biosynthesis of papaverine. A similar phenomenon was also observed in the aporphine pathway. Although some compounds, such as (S)-corytuberine and magnoflorine, have been reported in *C*. *yanhusuo*, we failed to find CYP80G2/CTS, key enzymes at the beginning of the aporphine pathway (green) [69].

### Analysis of functional genes and metabolites of the morphine pathway and phthalideisoquinoline pathway

Previous studies have shown that there are six specific biosynthetic enzymes involved in the morphine pathway (blue) [69]. However, only three functional enzymes were annotated in our transcriptome, namely, salutardine reductase (SalR), codeine reductase (COR2) and codeine O-demethylase (CODM). One single unigene was annotated as SalR. To verify the reliability of the annotation, we blastp in the NCBI database and obtained a hit (KAF5191451.1) with high similarity (61.513%) (S7 Table). We found nine unigenes encoding COR2 in the tblastn results, and only four unigenes had complete sequences. In the NCBI database, the blastp results of four unigenes (COR2) matched some homologues, which were annotated as nonfunctional NADPH-dependent codeinone reductase-2 in the Nr database, and the similarity was up to 69% (S7 Table). Unfortunately, the functional enzyme CODM only found one unigene with incomplete sequences and failed to obtain hits in the blastp results, so the reason for annotation as codeine O-demethylase was unclear. Maybe it was an incorrect annotation or functional interpretation of tblastn results, or an evolutionary genetic residue from other morphine producing plants. A previous study identified a series of genes mapped to the morphine pathway in *C*. *yanhusuo* bulbs [31]. In contrast, only COR and SalR were identified in our study, indicating that *C*. *yanhusuo* lacked the enzymes related to the biosynthesis of the morphine pathway, so we were unable to find the corresponding morphine, which was consistent with the chemical characteristics of nonaddiction about *C*. *yanhusuo* [2]. Similarly, no relevant metabolite in the phthalideisoquinoline pathway was found in *C*. *yanhusuo* bulbs. Although allocryptopine, a starting substance for the synthesis of noscapine, was detected, it is probably related to the structural similarity of protoopioid [21]. Noscapine and its encoding genes exist in all plant organs but are most abundant in stems and immature seed capsules [61]. Noscapine synthase (NOS), as the last enzyme in this BIAs pathway, is the only functional enzyme found in latex [62, 63]. Similarly, the formation of thebaine was mainly catalysed by enzymes located in phloem sieve elements, while T6ODM, COR and CODM mainly occurred in laticiferous [70], which may also be the reason why *C*. *yanhusuo* detected no metabolites related to the morphine pathway and noscapine pathway.

### Difference analysis of BIAs in suborgans and development stages of the *C*. *yanhusuo* bulb

Research has shown that the accumulation of secondary metabolites in plants dynamically changes due to different tissues and organs and developmental stages [71]. Previous reports demonstrated that the biosynthesis of BIAs in *C*. *yanhusuo* was space specific and mainly accumulated in roots rather than leaves [21]. Meanwhile, THP accumulated throughout the development stages of the bulb [31]. In this study, there was no significant difference in BIAs content or gene expression level between the two suborgans of the *C*. *yanhusuo* bulb (mother-bulb and son-bulb). This phenomenon may be explained by the fact that although the two suborgans were different in formation site, there was no genetic differentiation during the phylogenetic process in essence, so BIAs synthesis genes were regulated by the same genetic pattern, resulting in no significant differences in the expression levels of genes and metabolite accumulation. Second, the expression level of BIAs genes was highly active in the expansion stage (MB-A and SB-A), which was significantly higher than that in the maturity stage (MB-C and SB-C), and the content of BIAs was consistent with gene regulation. The possible reason for this result was that the aboveground parts grew luxuriantly during the expansion period, and primary metabolites accumulated by photosynthesis began to transfer to secondary metabolites, promoting the rapid accumulation of BIAs. However, at the maturity stage of the bulb, as the aboveground parts of the plant began to wither and die and the environmental conditions changed (high temperature), photosynthesis was weakened, leading to the synthesis of secondary metabolites being reduced or stopped. The content of berberine reached its highest in July in *Coptis chinensis*, showing a downwards trend with the passage of time [72], indicating that the content of active components of medicinal plants has dynamic changes due to different development times. The accumulation of metabolites is the result of genetic material regulation, and to some extent, they should be consistent.

## Conclusion

This study was devoted to constructing the biosynthetic pathway of BIAs and identifying candidate genes in *C*. *yanhusuo* bulbs based on widely targeted metabolome and transcriptomic analysis. Protoberberine-type and aporphine-type alkaloids have been indicated to be the dominant alkaloids in *C*. *yanhusuo* bulbs. Key genes involved in BIAs synthesis pathway include *6-OMT*, *CNMT*, *NMCH*, *BBE*, *SOMT1*, *CFS*, *SPS*, *STOX*, *MSH*, *TNMT* and *P6H*. There was no significant difference in the content of BIAs and the expression level of genes between the two suborgans (mother-bulb and son-bulb). The expression levels of BIAs genes in the expansion stage (MB-A and SB-A) were significantly higher than those in the maturity stage (MB-C and SB-C), and the content of BIAs was consistent with the pattern of gene regulation. Five complete single genes were likely to encode the functional enzyme of CoOMT and participated in tetrahydropalmatine (THP) biosynthesis in *C*. *yanhusuo* bulbs. These studies provided a strong theoretical basis for the subsequent development of metabolic engineering of BIAs (especially THP) of *C*. *yanhusuo*.

## Supporting information

S1 Fig Summary of BIAs pathway and heat-map of candidate genes of *C*. *yanhusuo* bulb. (PDF)

S2 Fig Content of nine compounds in bulb of *C*. *yanhusuo* by HPLC. (PDF)

S1 Table Overview of alkaloid metabolites in bulb of *C*. *yanhusuo*. (XLSX)

S2 Table Summary of sequence analyses. (PDF)

S3 Table Length abundance statistics of transcripts and unigenes. (PDF)

S4 Table The number of differentially expressed unigenes annotated to KEGG pathways. (XLSX)

S5 Table Secondary metabolic pathway analysis about top 20 of KEGG pathway. (PDF)

S6 Table Candidate genes sequence of O-methyltransferase for phylogenetic analysis. (PDF)

S7 Table Blastp result of *C*.*yanhusuo* unigenes of morphinan pathway. (XLSX)

## Abbreviations

3-OHase: tyrosine 3-hydroxylase
4’-OMT: (S)-3′-hydroxy-N-methylcoclaurine-4′-O-methyltransferase
6-OMT: (S)-norcoclaurine 6-O-methyltransferase
AT1: (13S,14R)-1,13-dihydroxy-Nmethylcandine 13-O-acetyltransferase
BBE: berberine bridge enzyme
BIAs: benzylisoquinoline alkaloids
CAS: canadine synthase
CFS: cheilanthifoline synthase
CNMT: (S)–coclaurine-N-methyltransferase
CODM: codeine-O-demethylase
CoOMT: columbamine O-methyltransferase
COR: codeinone reductase
CXE1: 3-O-acetylpapaveroxine carboxylesterase
DAM: differentially accumulated metabolite
DBOX: dihydrobenzophenanthridine oxidase
DEG: differentially expression gene
MB-A: mother-bulb expansion period
MB-C: mother-bulb maturity period
MSH: methylstylopine hydroxylase
N7OMT: norreticuline 7-O-methyltransferase
NCS: norcoclaurine synthese
NMCH: (S)-N-methylcoclaurine-3′-hydroxylase
NOS: noscapine synthase
P6H: protopine-6-hydroxylase
SalAT: salutaridinol-7-Oacetyltransferase
SalR: salutaridine reductase
SalSyn: salutaridine synthase
SB-A: son-bulb expansion period
SB-C: son-bulb maturity period
SOMT: scoulerine 9-O-methyltransferase
SPS: stylopine synthase
STORR: 1,2-dehydroreticuline synthase/reductase
STOX: (S)-tetrahydroxyprotoberberine oxidase
T6ODM: thebaine-6-O-demethylase
THP: tetrahydropalmatine
TNMT: tetrahydroprotoberberine-N-methyltransferase
TyDC: tyrosine decarboxylase
TyrAT: tyrosine aminotransferase
