# Supplementary figures and images for: Metabolomics and transcriptomics reveal the mechanism of alkaloid synthesis in Corydalis yanhusuo bulbs
Source: PLoS One. 2024 May 23;19(5):e0304258. doi: 10.1371/journal.pone.0304258 (PMC11115222; doi:10.1371/journal.pone.0304258)

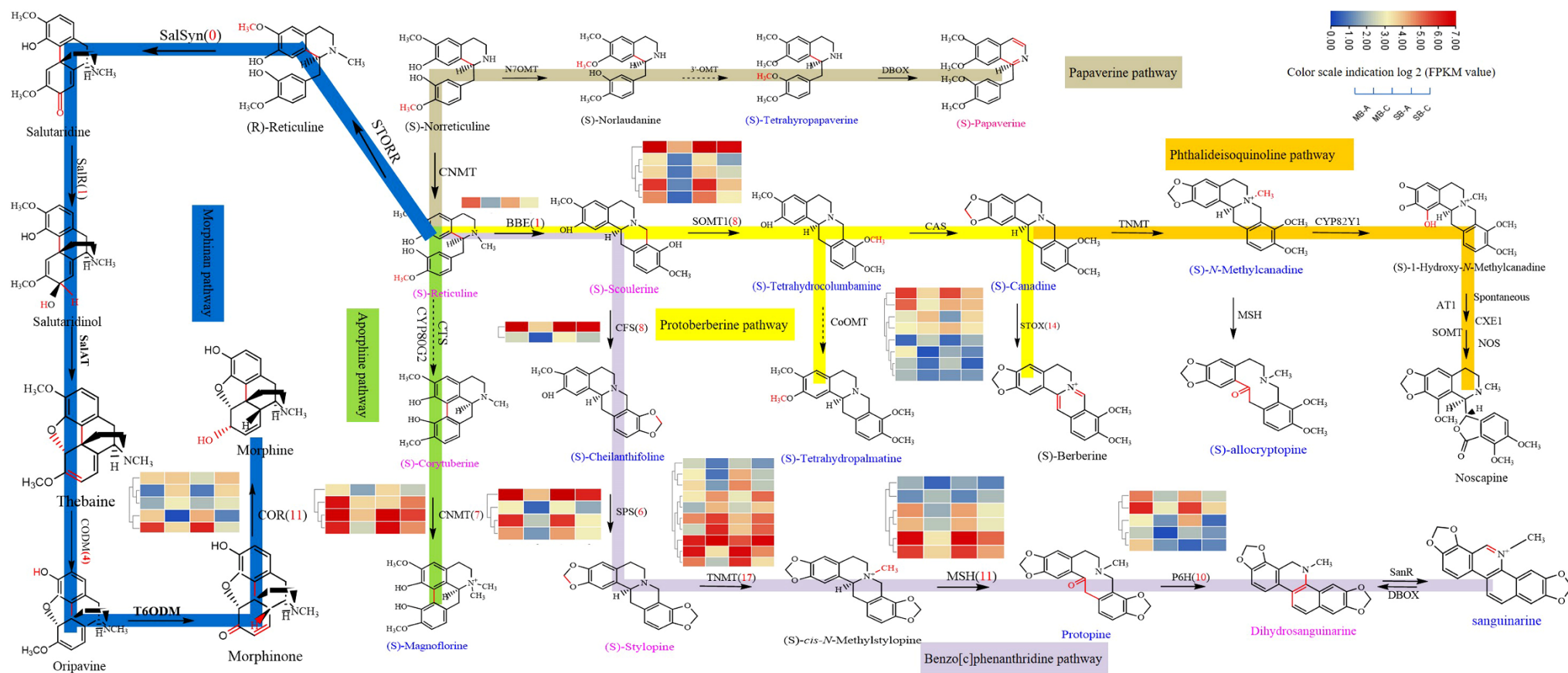

S1 Fig. Summary of BIAs pathway and heat-map of candidate genes of *C. yanhusuo* bulb.

Supplement: S1 Fig — (PDF) [file pone.0304258.s001.pdf]

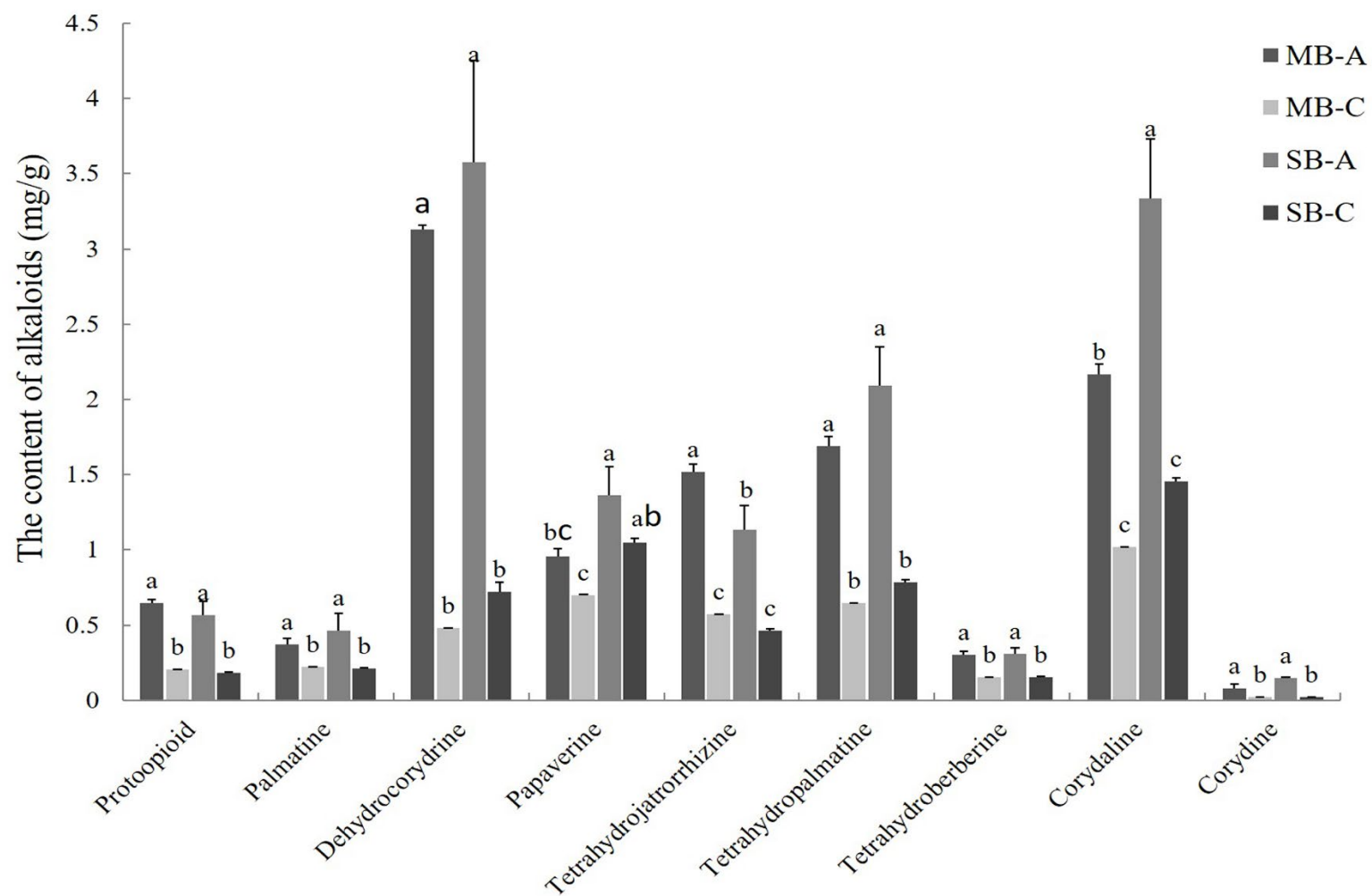

S2 Fig. Content of nine compounds in bulb of *Corydalis yanhushuo* by HPLC

Supplement: S2 Fig — (PDF) [file pone.0304258.s002.pdf]
