# Supplementary material for: Metabolomics and transcriptomics reveal the mechanism of alkaloid synthesis in Corydalis yanhusuo bulbs
Source: PLoS One. 2024 May 23;19(5):e0304258. doi: 10.1371/journal.pone.0304258 (PMC11115222; doi:10.1371/journal.pone.0304258)
Supplement: S2 Table — (PDF) [file pone.0304258.s004.pdf]

S2 Table. Summary of sequence analyses

Sample	Raw Reads	Clean Reads	Clean Base(G)	Error Rate(%)	Q20 (%)	Q30 (%)	GC Content(%)
MB-A1	69806188	68236742	10.24	0.03	97.21	92.77	42.86
MB-A2	73121966	70908406	10.64	0.03	97.34	93.06	42.83
MB-A3	70461930	68746576	10.31	0.03	97.28	92.91	43.07
MB-C1	94549664	91945556	13.79	0.03	97.32	92.83	43.08
MB-C2	95149584	92889100	13.93	0.03	97.28	92.77	43.05
MB-C3	88514622	86923748	13.04	0.03	97.26	92.68	42.95
SB-A1	107303986	103703094	15.56	0.03	97.48	93.25	43.07
SB-A2	93040398	90520784	13.58	0.03	97.42	93.1	43.11
SB-A3	94696712	92033112	13.8	0.03	97.35	92.93	42.91
SB-C1	107964730	104821144	15.72	0.03	97.4	93.04	42.97
SB-C2	94041224	91969634	13.8	0.03	97.32	92.84	42.87
SB-C3	93403848	91420136	13.71	0.03	97.38	92.97	42.94
