# Supplementary material for: Metabolomics and transcriptomics reveal the mechanism of alkaloid synthesis in Corydalis yanhusuo bulbs
Source: PLoS One. 2024 May 23;19(5):e0304258. doi: 10.1371/journal.pone.0304258 (PMC11115222; doi:10.1371/journal.pone.0304258)
Supplement: S3 Table — (PDF) [file pone.0304258.s005.pdf]

S3 Table. Length abundance statistics of transcripts and unigenes

Type	Transcript	Unigene
Total Bases	169521813	167915699
Number	190269	183631
Mean Length	891	914
N50	1431	1449
N90	361	374
200~300	39582	33384
300~400	31986	31640
400~500	20535	20476
500~600	14123	14103
600~700	10412	10408
700~800	8455	8454
800~900	6879	6876
900~1000	5903	5901
1000~1100	5207	5207
1100~1200	4552	4550
1200~1300	4096	4096
1300~1400	3668	3668
1400~1500	3249	3248
1500~1600	2899	2899
1600~1700	2632	2632
1700~1800	2290	2290
1800~1900	2211	2211
1900~2000	2019	2019
>=2000	19561	19561
