# Supplementary material for: Metabolomics and transcriptomics reveal the mechanism of alkaloid synthesis in Corydalis yanhusuo bulbs
Source: PLoS One. 2024 May 23;19(5):e0304258. doi: 10.1371/journal.pone.0304258 (PMC11115222; doi:10.1371/journal.pone.0304258)
Supplement: S5 Table — (PDF) [file pone.0304258.s007.pdf]

S5 Table. Secondary metabolic pathway analysis about top 20 of KEGG pathway

Group	KEGG pathway enrichment	Rich_factor	Qvalue	Gene number
MB-A vs MB-C	Isoquinoline alkaloid biosynthesis	0.203501	6.15E-12	93
	Monoterpenoid biosynthesis	0.208333	0.001247	15
	Flavonoid biosynthesis	0.147651	0.010632	22
	Flavone and flavonol biosynthesis	0.294118	0.012953	5
SB-A vs SB-C	Isoquinoline alkaloid biosynthesis	0.227571	1.27E-07	104
	Flavone and flavonol biosynthesis	0.529412	0.000153	9
SB-A vs MB-A	Anthocyanin biosynthesis	0.166667	0.000538	2
	Flavone and flavonol biosynthesis	0.117647	0.001099	2
SB-C vs MB-C	Isoquinoline alkaloid biosynthesis	0.026258	2.11E-07	12
	Indole alkaloid biosynthesis	0.05	0.010392	2
	Flavone and flavonol biosynthesis	0.058824	0.063401	1
