# Supplementary material for: Metabolomics and transcriptomics reveal the mechanism of alkaloid synthesis in Corydalis yanhusuo bulbs
Source: PLoS One. 2024 May 23;19(5):e0304258. doi: 10.1371/journal.pone.0304258 (PMC11115222; doi:10.1371/journal.pone.0304258)
Supplement: S6 Table — (PDF) [file pone.0304258.s008.pdf]

S6 Table. Candidate genes sequence of O-methyltransferase for phylogenetic analysis

>6OMT Papaver somniferum

METVSKIDQQNQAKIWKQIYGFAESLVLKCAVQLEIAETLHNNVKPMSLSELASKLPVAQPVNEDRLFRIMRYLV
HMELFKIDATTQKYSLAPPAKYLLRGWEKSMVDSILCINDKDFLAPWHHLGDGLTGNCDAFEKALGKSIWVYM
SVNPEKNQLFNAAMACDTRLVTSALANECKSIFSDGISTLVDVGGGTGTAVKAISKAFPDIKCTIYDLPHVIADSP
EIPNITKISGDMFKSIPSADAIFMKCILHDWNDDECIQILKRCKEALPKGGKVIIVDVVIDMDSTHPYAKIRLTLDL
DMMLNTGGKERTKEEWKTLFDAAGFASHKVTQISAVQSVIEAYPY

>6OMT Thalictrum flavum subsp. glaucum

MEMINKENLSSQAKLWNFIYGFADSLVLKSAVQLDLANIIHNHGSPMTLSELSLHLPSQPVNQDALYRVLRYLVH
MKLFTKSSIDGELRYGLAPPAKFLVKGWDKCMLGAILTITDKDFMAPWHYLKEGILNDGSTSTAFEKALGTNIW
DYMAEHPEKNQLFNEGMANDTRLIMSALVKECSSMFDGITTIVDVGGGTGTAVRNIAKAFPHIKCTVYDLPHVI
ADSPGYTEINSIQGDMFKYIPNADAIMMKCILHDWDDKECIEILKRCKDAVPRDGGKVIIIDIILDVKSEHPYTKM
RLTLDLDMMLNTGGKERTEEEWKKLIHDAGYKGYKITHISAVQSVIEAYPY

>6OMT Sinopodophyllum hexandrum

MEAQKENISSQAKLWNFIYGFAESLVLKCAVELDFANIIHNHGKPMTLSELASQLPVMQPVNTNSLYRVMRYLV
HINIFTKTLDENDGETKYGLAAPAKFLVKGWDNCMVGSILGITDKVFMEPWYYIKDELAPGTGTAFELALGKDI
WEYMGENPEKNKLFNAAMACDTSMIMSALISECKDKFNGIRTLVDVGGGTGTAARNIARAFPNIKCTVYDLPH
VIADSPVYPEINRVSGDMFKCIPNADAILMKCILHDWEDKECIEILKRCKEAVPVDGGKVIIIDVVLDGESEHPYT
KVRLNSDLDMMLNTEGKERTKEGWKKLFKAAGYRDYNITQISALQSVIEAFPY

>6OMT Thalictrum thalictroides

MIKKENLSSQAKLWNIIYGFAESLVLKSAVQLDLANIIHDHGTPMTLSELSLCLPSQPVNQDALYRIMRYLVHMKL
FTKSSIDGELRYGLAPPAKFLVKGWDNCMVGAILTISDKEFMAPWHYLKDGLSSDGGTSTAFEKALGSNIWEYM
AEHPEKNQIFNEGMANDSRLIMSALVKECSSMFEGITTLVDVGGGTGTAVRNIAKAFPHIKCTVYDLPHVIADA
PAGYTEINFVQGDMFKYIPNADAITMKCILHDWDDNECIEILKRCKEAVPRDGGKVIIIDIILDVKSEHPYTKMRL
TLDLDMMLNTGGKERTEEEWKKLIYDAGYKGYKITHISAVQSVIEAYPY

>6OMT Coptis japonica

MEVKKDNLSSQAKLWNFIYGFAESLVLKCAVQLDLANIIHNSGTSMTLSELSSRLPSQPVNEDALYRVMRYLVH
MKLFTKASIDGELRYGLAPPAKYLVKGWDKCMVGSILAITDKDFMAPWHYLKDGLSGESGTAFEKALGTNIWG
YMAEHPEKNQLFNEAMANDSRLIMSALVKECGNIFNGITTLVDVGGGTGTAVRNIANAFPHIKCTVYDLPHVIA
DSPGYSEVHCVAGDMFKFIPKADAIMMKCILHDWDDKECIEILKRCKEAVPVKGGKVIIVDIVLNVQSEHPYTK
MRLTLDLDMMLNTGGKERTEEEWKKLIHDAGYKGHKITQITAVQSVIEAYPY

>6OMT Papaver bracteatum

METVSKIDQQNQAKIWKQIYGFAESLVLKCAVQLEIAETLHNNVKPMSLSELASKLPVPQPVNEDRLFRIMRYLV
HMELFKIDATTQKYSLAPPAKYLLRGWEKSMVDSILCINDKDFLAPWHHLGDGLTGNCDAFEKALGKSIWVYM
SENPEKNQLFNAAMACDTRLVTSALANECKSIFSDGIKTLVDVGGGTGTAVKTISKAFPDIKCTIYDLPHVIADSPE
IPNITKISGDMFKSIPSADAIFMKCILHDWNDEECIQILKRCKEALPKDGKVIIVDVVIDMDSTHPYAKIRLTLDLD
MMLNTGGKERTKEEWKILFDAAGFASHKVTQISAVQSVIEAYPY

>Cluster-3224.83636

MEGMSDLSLENEAVTIWKFIYGFCDTLVLKCCVNLEIADTIHKHGQPMTLSELASQLSVDLQKTIDTDRLYRLMR
YLVHLKFFTTEEGSDHLGEIKYGLLPLAKFLIRGQKSMAGLITVVDKDFIAPWHHLEDGLDGRTDAFEVAFGKKV
FDYLSENPESNRRFNEYMASFSSLVALELVNCNNVFEDRIKTLVDAGGGTGTTAKAIANAFPHIKCMVYELPHVN
ADAPVDPNIQRIDGDIFKSVPKADAILMQGVLHDWNDDECIQILKNCRESIPQDGGKVILIEVVVNANSNHPYA
KLMLLADLEMVIYGGKERTDEEWKKLLEAAGFTRYKLTEISAMHSIIEAYPY*

>Cluster-3224.92563

MEVIKKSDQTDQAKLWKFIYGFADSLVLKCAVELEIADTIHKHGEPMTLSELASQLPKQPIDADRLYRIMRYLVQI
KLFSKETTSQSGEIKYGLLPPAKYVVRGWQNSMVAALLLINDKNFIASWHYLKDGLGGECDAFEKANGKKIWD
FMSENPEKNKLFNEAMACDSRLVTWALVQDCKDVFKGIKTLVDVGGGTGTAVKAISDAFPDIKCAVYDLPHVIA
DSPVAPNIDRIEGDMFKSIPNADAIFMKCILHDWNDDECIQILKQCKKALPRDGGKVIIVDVVLNVDSKHPYTK
MRLTLDLDMMLNTGGKERTEEEWKELFEAAGFSGYKIIQTSALQSVIEAYP*
>Cluster-3224.102293
MEVIKKSDQTDQAKLWKFIYGFADSLVLKCAVELEIADTIHKHGEPMTLSELASQLPKQPIDADRLYRIMRYLVQI
KLFSKETTSQSGEIKYGLLPPAKYVVRGWQNSMVAALLLINDKNFIASWHYLKDGLGGECDAFEKANGKKIWD
FMSENPEKNKLFNEAMACDSRLVTWALVQDCKDVFKGIKTLVDVGGGTGTAVKAISDAFPDIKCAVYDLPHVIA
DSPVAPNIDRIEGDMFKSIPNADAIFMKCILHDWNDDECIQILKQCKKALPRDGGKVIIVDVVLNVDSKHPYTK
MRLTLDLDMMLNTGGKERTEEEWKELFEAAGFSGYKIIQTSALQSVIEAYP*
>Cluster-3224.83637
MEGMSDLSLENEAVTIWKFIYGFCDTLVLKCCVNLEIADTIHKHGQPMTLSELASQLSVDLQKTIDTDRLYRLMR
YLVHLKFFTTEEGSDHLGEIKYGLLPLAKFLIRGWPKSMAGLLTAMDKDFIAPWHHLEDGLDGRTDAFEVAFGK
KVFDYLSENPKSSQLFNDYMAIHSSLLALELVKCKNVFEDRIKTLVDAGGGTGTTAKAIANAFPHIKCMVYELPHV
NADAPVDPNIQRIDGDIFKSVPKADAILMQGVLHDWNDDECIQILKNCRESIPQDGGKVIIIEVVVNANSNHPY
TKIMLLADLDMLIHGGRERTDEEWKKLLEAAGFTRYKLTEISAMHSIIEAYPY*
>Cluster-3224.64109
MACVAKAYPHVKCKSFDLPHVVAEAPAFPGVELFGGDMFEFIPPADAISLKSFLHGSQDEECIKLLKRCKEVIPAD
KGKVIIIEIVLDQDEDDDELTTARVGLDIDTMLSSEGKERTKDEWRVLVEKAGYSRVEIIPIFAIPSVIVAYP*
>Cluster-3224.80789
MEITINGGNNEQEMKWQGQIWSHICGIVDSIVLQTTLELNIFDTVHTHTNSRISFSQLSKSPSLASIKPQNLYRM
LRYLVHLNLLAIKVIEGEETFSLTNLSKLLLENQEKSLRDWSLGVNHQDLIDPWHELSKFVTDPADAPTPFVQIHG
KTFWEWSGENPELNALINNTMASDSRLVMPAVVQGCQELFNGISSLVDIGGGTGTAMACVAKAYPHVKCKSF
DLPHVVAEAPAFPGVELFGGDMFEFIPPADAISLKSILHSWQDEACIKLLKRCKEVIPADKGKVIIIEIVLDQDEDD
DELTKARVSLDIDMMMNAGGKERTKDEWRVLVEKAGYSRVEIIPIFAIPSVIVAYP*
>Cluster-3224.64108
MEITINGGNNEQEMKWQGQIWSHICGIVDSIVLQTTLELNIFDTVHTHTNSRISFSQLSKSPSLASIKPQNLYRM
LRYLVHLNLLAIKVIEGEETFSLTNLSKLLLENQEKSLRDWSLGVNHQDLIDPWHELSKFVTDPADAPTPFVQIHG
KTFWEWSGENPELNALINNTMASDSRLVMPAVVQGCQELFNGISSLVDIGGGTGTAMACVAKAYPHVKCKSF
DLPHVVAEAPAFPGVELFGGDMFEFIPPADAISLKSILHSWQDEACIKLLKRCKEVIPADKGKVIIIEIVLDQDEDD
DELTKARVSLDIDMMMNAGGKERTKDEWRVLVEKAGYSRVEIIPIFAIPSVIVAYP*
>Cluster-3224.119047
MEITINGGNNEQEMKWQGQIWSHICGIVDSIVLQTTLELNIFDTVHTHTNSRISFSQLSKSPSLASIKPQNLYRM
LRYLVHLNLLAIKVIEGEETFSLTNLSKLLLENQEKSLRDWSLGVNHQDLIDPWHELSKFVTDPADAPTPFVQIHG
KTFWEWSGENPELNALINNTMASDSRLVMPAVVQGCQELFNGISSLVDIGGGTGTAMACVAKAYPHVKCKSF
DLPHVVAEAPAFPGVELFGGDMFEFIPPADAISMKSILHSRQDETCIQLLKRCKEVIPEDKGKVIIDIVLDQDEDD
DELTKARVSLDIDMMLSSGGKERTKEEWRVLVEKSGYSRVEIIPIFAIPSVIVAYP*
>Cluster-3224.105742
MEITINGGNNEQEMKWQGQIWSHICGIVDSIVLQTTLELNIFDTVHTHTNSRISFSQLSKSPSLASIKPQNLYRM
LRYLVHLNLLAIKVIEGEETFSLTNLSKLLLENQEKSLRDWSLGIDNKTSIDTWHELSNVVTDPADAPTPFVQIHGK
TFWEWSGENPELNALINNTMASDSRLVMPAVVQGCQELFNGISSLVDIGGGTGTAMACVAKAYPHVKCKSFD
LPHVVAEAPAFPGVELFGGDMFEFIPPADAISMKSILHSRQDETCIQLLKRCKEVIPEDKGKVIIID
>Cluster-3224.80788

PRNPKQPGKKILSHSKFLDFQEMEITINGGNNEQEMKWQGQVWSHICGFVDSTVLKTTLELSIFDTVHTHTNS
LITFSQLSKSPFLASIKPQNLYRMLRYLVHLNLLTIKVVEGEETFSLTILSKLLLENKDKSLRDWSLGIDDPTSINIWHE
LSKFVTDPADAPTPFVQIHGKTLWELAGEIPEVNTLINNAMACDTRLVMPAVVQGCQELLNGISSLVDIGGGTG
TAMACVAKAYPHVKCKSFDLPHVVAEAPAFPGVELFGGDMFEFIPPADAISLKFMLHNWLDEACIKLLKRCKEVI
PEDKGKVIIIDIVLDQDEDDDELTKARVSLDIDMMLSSGGKERTKEEWRVLVEKSGYSRVEIIPIFAIPSVIVAYP*
>4'-OMT2 Papaver somniferum]
MGSLDAKPAAATQEVSIKDQAQLWNIIYGFADSLVLRCAVEIGIADIIKNNDGAITLAQLAAKLPITNVSSDYLYR
MVRYLVHLNIIEQETCNGGVEKVYSLKPVGTLLLRDAERSMVPMILGMTQKDFMVSWHFMKEGLGNGSTTAF
EKGMGMDIWKYLEGNPDQSQLFNEGMAGETRLLTKTLIEDCRDTFQGLDSLVDIGGGNGTTIKAIYEAFPHIKC
TLYDLPHVVANSHDLPNIEKVPGDMFKSVPSAQAILLKLILHDWTDEECVNILKKCKEAIPKETGKVIIVDVALEEE
SNHELTKTRLILDIDMLVNTGGRERTADDWENLLKRAGFRSHKIRPIRAIQSVIEAFP
>4'-OMT Coptis japonica
MAFHGKDDVLDIKAQAHVWKIIYGFADSLVLRCAVELGIVDIIDNNNQPMALADLASKLPVSDVNCDNLYRILR
YLVKMEILRVEKSDDGQKKYALEPIATLLSRNAKRSMVPMILGMTQKDFMTPWHSMKDGLSDNGTAFEKAM
GMTIWEYLEGHPDQSQLFNEGMAGETRLLTSSLISGSRDMFQGIDSLVDVGGGNGTTVKAISDAFPHIKCTLFD
LPHVIANSYDLPNIERIGGDMFKSVPSAQAIILKLILHDWNDEDSIKILKQCRNAVPKDGGKVIIVDVALDEESDH
ELSSTRLILDIDMLVNTGGKERTKEVWEKIVKSAGFSGCKIRHIAAIQSVIEVFP
>4'-OMT Thalictrum flavum subsp. Glaucum
MAAFQGKDASVDIKAQAHVWKIIYGFADSLVLRCAVELGIFDIIDNNNQPIPLADLASKLPISNVNLDNLFRILRYL
VKMELLSYAADDKYALEPIAKLVLRNEKRSMVPMVLGMTQKDFMTPWHSMKDGLTDNGVTAFEKAMGMTI
WEYLEGHPDQSQLFNEGMAGETRLLTSSLISGSKDMFQSIDSLVDVGGGNGTTIKAISVAFPHIKCTLFDLPHVI
ANSYDHPNIERIGGDMFKGMPSAQAILLKLILHDWNDEDSIKILKQCRKAVPKDGGKVIIVDVALDEDSDHELSS
TRLILDIDMLVNTGGKERTKEDWEKLVKCAGFSGCKIRHIAAIQSVIEVFP
>4'-OMT Eschscholzia californica
MGLEFNEEVDIKAQAHLWNIIYGFADSLVLRSAVELGIADIIKNNNGSITVSELASKLPISNVNSDNLYRVLRYLVH
MGILKETKSTINGGEIKKLYSLEPVGSLLVKDAERNMVPIVLGMTQQDFMIPWHYIKEGLGEGSTAFEKGMGM
TLWEYLEGHPEQGHLFNVGMEGETRLLTKTLIESCKDTFEGLSSLVDVGGGNGTTIKAISEAFPHIKCSLYDLPHV
VADSHDLPNIEKIPGDIFKFIPNAQAILLKLILHDWSDEDSVKILKKCREAVPQDTGRVIIVDVALEEESEHPLTKTRL
VLDVDMLVNTGGRERSEDDWAKLLKLAGFRTHKIRHIAAVQSVIEAFP
>Cluster-3224.93089
MGVNDIAEAQDVDIKAQAHLWNIIYGFADSLVLRCAVELGIADIINSNNGTVTISDIASKLPVDNVNEENLYRVLR
YLVYMGLLKESQDKCYSLEPVATLLLKDAQRSMVPIILGMTQKDFMVPWFFMKEGLGSGSTTAFEKGMGMTL
WEYLEGHPDQSQLFNEGMAGETRLLTSSLINGCRDTFQGLTSLVDVGGGNGTTIKGIYDAFPHIKCSVYDLPHVI
ANAHPHPNIERIPGDMFKSVPSAQAILLKLILHDWTDEESVDILKRCREAVPKEGGRVIIVDVALEEGSEHELTKT
RLILDIDMLVNTGGRERTVDDWDRMLKLAGFSSHKIRHIAAIQSVIEAFP*
>SOMT1 Coptis japonica
MCTSLSELKCPVFSTKRKLLLEFALRTSVDMAAQEGVNYLSGLGLSRLICLPMALRAAIELNVFEIISQAGPDAQLS
PSDIVAKIPTKNPSAAISLDRILRMLGASSILSVSTTKSGRVYGLNEESRCLVASEDKVSVVPMLLFTSDKAVVESFY
NIKDVVLEEGVIPFDRTHGMDFFQYAGKEERVNKSFNQAMGAGSTIAFDEVFKVYKGFDNLKELVDVGGGIGT
SLSNIVAKHPHIRGINFELPHVIGDAPDYPGVEHVPGDMFEGVPNAQNILLKWVLHDWDDDRSIKILKNCWKA
LPENGTVIVIEFVLPQVLGNNAESFNALTPDLLMMALNPGGKERTTIEFDGLAKAAGFAETKFFPISQGLHVME
FHKINC
>SOMT1 Papaver somniferum
MATNGEIFNTYGHNRQTATVTKITASNESSNGVCYLSETANLGKLICIPMALRAAMELNVFQLISKFGTDAKVSA

SEIASKMPNAKNNPEAAMYLDRILRLLGASSILSVSTTKKSINRGGDDVVVHEKLYGLTNSSCCLVPRQEDGVSLV
EELLFTSDKVVVDSFFKLKCVVEEKDSVPFEVAHGAKIFEYAATEPRMNQVFNDGMAVFSIVVFEAVFRFYDGFL
DMKELLDVGGGIGTSVSKIVAKYPLIRGVNFDLPHVISVAPQYPGVEHVAGDMFEEVPKGQNMLLKWVLHDW
GDERCVKLLKNCWNSLPVGGKVLIIEFVLPNELGNNAESFNALIPDLLLMALNPGGKERTISEYDDLGKAAGFIK
TIPIPISNGLHVIEFHK
>SOMT1 Thalictrum flavum subsp. Glaucum
MALQEGVNYLSGLGLSRLICLPMALRAAIELNVFEIIFQAGPEAQLSPAEIVAKIPTKNPNAAIALDRILRMLGASS
ILSVTTMKDGRVYGLTEESRCLVADKNGVSVVPMLLFTSDKAVVESFYNIKDVVLEEGVIPFDRTHGMDFFAYAG
KEQSVNKSFNQAMGAGSTIAFDEVFKVYKGFHDLKELVNVGGGIGTSLSNIIFKYPHIKGINFELPHVIADAPNYP
GVEHIAGNMFEGVPNAQNILLKWVLHDWDDERSIKILQNCWKALPEGGTVIVVEFVLPQILGNNAESFNALTP
DLLMMTLNPGGKERTTTEFDGLAKAAGFAETKFFPISQGLHVMEFHKATAGVAS
>SOMT1 partial-Stephania intermedia
EGANFLAGLGSLGRLICIPMALKAALDLQVFEIIASAGAGSHLSASEIASRIQTTNPNAATALDRILRMLAAASLLS
VTTRPNHSTSERAYGLTPETLSLVPNKEGVSAAAMMRLVTDEAVIKSFCGLKHAVVEEGCVPFDKLHGENFFKYA
AKESRVNQVFNEAMAAGSAIAFEEVFKVYEGFKEVRELVDVGGGVGTSLGRIVGRYPHISGINFELSHVIDGAPT
YPGMKHVAGDMFEGIPNAQTIMLKWVLHDWGDEYCEKILKNCWKALPGEGGKVIVVEFVLPEELGNNAETF
NALIPDLLMMSLNPGGKERTLAQYEDLAMATGFSKTKAFPISLGLHVLEFLK
>Cluster-3224.83418
MDYKAQPVNYLSGFELLSRLACFPMALRAAIDLNVFQIISRFGPDAKLNSSQLVAEMPTTNPNAASALERILRIL
AANSLLSPSNELNGEISYGLTKDSRYLIPDQKDGVSLVPMVLLSINKYVMESFFQLKDAVLDEGCVPFDRTFGVSI
FEFAGKEPKVGKMFNEAMRSSSIYVLDEVLKVYEGFDEMKELVDVGGGIGGTMSKIVSKFSHIHGINFDLPHVI
ADAPSYPGVKHISGDMFEEIPKAENIFLKV*
>Cluster-3224.94041
MDYKAQPVNYLSGFELLSRLACFPMALRAAIDLNVFQIISRFGPDAKLNSSQLVAEMPTTNPNAASALERILRIL
AANSLLSPSNELNGEISYGLTKDSRYLIPDQKDGVSLVPMVLLSINKYVMESFFQLKDAVLDEGCVPFDRTFGVSI
FEFAGKEPKVGKMFNEAMRSSSIYVLDEVLKVYEGFDEMKELVDVGGGIGGTMSKIVSKFSHIHGINFDLPHVI
ADAPSYPGVKHISGDMFEEIPKAENIFLKWVLHDWDDESCKKLLRKCWNALDEGGKVIVIELVLPEVLGNNAES
HSALAADLIMMALSPGGKERTIIQFHNLAQAAGFNIVKSFPVNQGLHVIEFQK*
>Cluster-3224.99164
MDYKAQPVNYLSGFELLSRLACFPMALRAAIDLNVFQIISRFGPDAKLNSSQLVAEMPTTNPNAASALERILRIL
AANSLLSPSNELNGEISYGLTKDSRYLIPDQKDGVSLVPMVLLSINKYVMESFFQLKDAVLDEGCVPFDRTFGVSI
FEFAGKEPKVGKMFNEAMRSSSIYVLDEVLKVYEGFDEMKELVDVGGGIGGTMSKIVSKFSHIHGINFDLPHVI
ADAPSYPGVKHISGDMFEEIPKAENIFLKWVLHDWDDESCKKLLRKCWNALDEGGKVIVIELVLPEVLGNNAES
HSALAGDLMMMALSPGGKERTIIQFHNLAQAAGFNIVKSFPVNQGLHVIEFQK*
>Cluster-3224.103137
MDYKAQPVNYLSGFELLSRLACFPMALRAAIDLNVFQIISRFGPDAKLNSSQLVAEMPTTNPNAASALERILRIL
AANSLLSPSNELNGEISYGLTKDSRYLIPDQKDGVSLVPMVLLSINKYVMESFFQLKDAVLDEGCVPFDRTFGVSI
FEFAGKEPKVGNIFNEAMRSSSSFVLDEVFKVYEGFDEMKELVDVGGGIGGTMSKIVSKFSHIHGINFDLPHVIV
DAPNYPGVKHISGDMFEEIPKAENIFLKV*
>Cluster-3224.108600
MCTYVPFGSCTQKNYIYLALCKVSYLLQWVLHDWDDESCKKLLRKCWNALDEGGKVIVIELVLPEVLGNNAESH
SALAADLIMMALSPGGKERTIIQFHNLAQAAGFNIVKSFPVNQGLHVIEFQK*
>Cluster-3224.90849
MDYKAQPVNYLSGFELLSRLACFPMALRAAIDLNVFQIISRFGPDAKLNSSQLVAEMPTTNPNAASALERILRIL

AANSLLSPSNELNGEISYGLTKDSRYLIPDQKDGVSLVPMVLLSINKYVMESFFQLKDAVLDEGCVPFDRTFGVSI
FEFAGKEPKVGKMFNEAMRSSSIYVLDEVLKVYEGFDEMKELVDVGGGIGGTMSKIVSKFSHIHGINFDLPHVI
ADAPSYPGVKHISGDMFEEIPKAENIFLKWVLHDWDDESCKKLLRKCWNALDEGGKVIVIELVLPEVLGNNAES
HSALAGDLMMMALSPGGKERTIIQFHNLAQAAGFNIVKSFPVNQGLHVIEFQK*
>Cluster-3224.97651
MDYKAQPVNYLSGFELLSRLACFPMALRAAIDLNVFQIISRFGPDAKLNSSQLVAEMPTTNPNAASALERILRIL
AANSLLSPSNELNGEISYGLTKDSRYLIPDQKDGVSLVPMVLLSINKYVMESFFQLKDAVLDEGCVPFDRTFGVSI
FEFAGKEPKVGKMFNEAMRSSSIYVLDEVLKVYEGFDEMKELVDVGGGIGGTMSKIVSKFSHIHGINFDLPHVI
ADAPSYPGVKHISGDMFEEIPKAENIFLKWVLHDWDDESCKKLLRKCWNALDEGGKVIVIELVLPEVLGNNAES
HSALAADLIMMALSPGGKERTIIQFHNLAQAAGFNIVKSFPVNQGLHVIEFQK*
>Cluster-3224.99288
MDYKAQPVNYLSGFELLSRLACFPMALRAAIDLNVFQIISRFGPDAKLNSSQLVAEMPTTNPNAASALERILRIL
AANSLLSPSNELNGEISYGLTKDSRYLIPDQKDGVSLVPMVLLSINKYVMESFFQLKDAVLDEGCVPFDRTFGVSI
FEFAGKEPKVGNIFNEAMRSSSIFVLDEVLKVYEGFDEMKELVDVGGGIGGTMSKIVSKFSHIHGINFDLPHVIV
DAPNYPGVKHISGDMFEEIPKAENIFLKWVLHDWDDESCKKLLRKCWNALDEGGKVIVIELVLPEVLGNNAES
HSALAGDLMMMALSPGGKERTIIQFHNLAQAAGFNIVKSFPVNQGLHVIEFQK*
>CoOMT Coptis japonica
MDTPNTFQNDDEIKAQAQVWKHMFGFAETIMLRSTVSLGIPDIIHNNGPVTLSQLVTHLPLKSTSIDRFHHFM
RYLVHMQLFTISTDQITKEDKYELTPASKLLVHGHQKSLAPYVMLQTHPEEFSVWSHVINVLDGKKPYWESNDT
SMYEKTEGDPEINEILNDAMTSHSTFMLPALVSGLMKENVLDGVASIVDVGGNSGVVAKGIVDAFPHVKCSVM
DLNHVIERVIKNPKLDYVAGDMFTSIPNADAILLKSTLHNYEDDDCIKILNIAKEALPSTGGKVILVEIVVDTENLPL
FTSARLSMGMDMMLMSGKERTKKEWEDLLRKANFTSHQVIPIMAIESIIVAYS
>CoOMT Thalictrum thalictroides
MSAITPNTVLKPFEKEEIKAQAQVWKHMFGFAETIMLRTIVSLGIPDIVHSHGPITLSQLATQLPIKSLSIDKLNHF
MRYVVHMKLLKISTDEITKESKYELTPASELLVKSHNKSLAPYVMLQTHPEEFSVWGHVVDCLEGKKSCWESTY
GVSVYATVEKSQEMYNDLVNDAMTSHTRIMVPAVVSGLMKEKVLDGIGSIVDVAGSSGVATKAIVDAFPHIKCS
VMDLSHVIDSVIKDPKLHYVAGDMFTSVPNADAIFLKSTLHNYGDDECLKILSNAKEAIPCAGGKVILVDIVVDIE
GLPEFCSARLSMEMEMMLMGGKERTKKEWETLLSKAGFSHHKIIPIVAIESIIVAYA
>N7OMT Papaver somniferum
MEVVSQIDQENQAIIWKQIYGFSESLLLKCAVQCEIAETIHNHGTPMSILELAAKLPIDQPVNIDRLYRVMRYLVH
QKLFNKEVISTLNGGTVQVTEKYWLAPPAKYLIRGSQQSMVPSVLGIIDEDMFAPWHILKDSLTGECNIFETALG
KSISVYMSENPEMNQISNGAMAFDSGLVTSHLVNECKSVFGDEIKTLVDVGGGTGTALRAISKAFPNIKCTLFDL
PHVIADSPEIPTITKVSGDMFKSIPSADAIFMKNILHDWNDDECIQILKRCKDVVSAGGKLIMVEMVLDEDSFHP
YSKLRLTSDIDMMVNNGGKERTEKEWEKLFDAAGFASCKFTQMSVGFAAQSIIEVY
>N7OMT like-Papaver somniferum
MEVVSQNDQENQAIIWQQIYGFSESLILKCAVQLEIAETIHHHGTPMSISELAAKLPIDQPVNMDRLYRVMRYL
VHMKLFNKEEIIYTHNGGTVEKYSLAPPAKYLIRGSERSMVPSILGTIHKDLLAAWDILKDSLTGNCNVFEKALGR
NISVYYSENLEMNKISNEAMAFDSGLFTSALVNECKSVFGDDIKTLVDVGGGTGTAIKAISMAFPNIKCTLFDLPH
VIDDSPETPTITKISGDMFKSIPSADAIFMKNILHDWNDDECIQILKRCKDAVSPGGKLIIVEMVMDMDSVHHP
YSKLRLASDMDMMVSNGGKERTEKEWKELFDPTGFACCKITQMSAGFAAQSVIEVFIDDVIEESGFAK
>N7OMT like2-Papaver somniferum
MEVVSQNDQENQAIIWNQIYGFSGSLLLKCAVQLKIAETIHNHGAPISISELAAKLPIDQPVNMDRLYRVMRYL
VHMKLFNKEIISTLNGGTIHVTEKYSLAPPAKYLIRGSQQSMVPAVLSITDKDIFSAWQILNDSLTGNCNVFEKAL
GKDISVYMSENPEMNENIRGAMAFNSGLITSALVKECKSVFGDEIKTLVDVGGGTGTVVTAISKAFPNIKCTLFD

LPHVIAISHDIPTVTKNSGDMFKSIPSADAIFMKNILHDWDDDEAIQILKRCKDVLSPGGKLIIVEMVLDVDSVH
PYSKLRLTSDIDMMVNTGGKERTEKEWKKLFDEAGFGSCKITQM
